# Pyroptosis in Peripheral Neuropathy: From Molecular Mechanisms to Therapeutic Targeting

**DOI:** 10.1002/cns.70760

**Published:** 2026-01-23

**Authors:** Jinhuan Wei, Mingyue Liuyuan, Zhixin Ye, Xueying Wang, Xueli Zhou, Yifan Shan, Cheng He, Chunting Zhu, Chicheng Zhou, Jingyin Bao, Yonghui Zhang, Gang Chen

**Affiliations:** ^1^ Center for Basic Medical Research Medical School of Nantong University, Co‐Innovation Center of Neuroregeneration Nantong Jiangsu China; ^2^ Department of Human Anatomy Shandong Medical College Linyi Shandong China; ^3^ Department of Medical Equipment Affiliated Hospital of Nantong University Nantong Jiangsu China; ^4^ Department of Anesthesiology Affiliated Hospital of Nantong University Nantong Jiangsu China; ^5^ Key Laboratory of Neuroregeneration of Jiangsu and the Ministry of Education, Coinnovation Center of Neuroregeneration Nantong University Nantong Jiangsu China

**Keywords:** gasdermin, inflammasome, molecular mechanisms, peripheral neuropathy, pyroptosis, therapeutic targets

## Abstract

**Background:**

Peripheral neuropathy (PN) is a common consequence of peripheral nervous system (PNS) disorders, yet its effective treatment remains a significant clinical challenge. Pyroptosis, an inflammatory form of programmed cell death (PCD) triggered by gasdermin A–E (GSDMA‐E), contributes to the pathogenesis of PN and represents a promising therapeutic target. While reviews of pyroptosis in other diseases are extensive, comprehensive reviews focusing on PN are lacking.

**Method:**

We systematically searched PubMed, Scopus, Web of Science, and Google Scholar (1986–2025). Only original studies investigating pyroptosis in PN were included.

**Results:**

This review first consolidates established evidence, highlighting a context‐dependent dual role of pyroptosis in PN. Its detrimental effects in chronic pain involve canonical (caspase‐1/GSDMD) or noncanonical pathways (e.g., caspase‐4/5/11/GSDMD, caspase‐3/GSDME, caspase‐8/GSDMC), often initiated by inflammasomes (e.g., NOD‐like receptor family pyrin domain containing 3 [NLRP3]). Conversely, its beneficial, tumoricidal role is leveraged in neuroblastoma. Preclinically, diverse inhibitors—including NLRP3 inhibitors (e.g., MCC950), caspase‐1 inhibitors (e.g., VX‐765), and P2X7R antagonists (e.g., Brilliant Blue G)—alleviate pain and promote nerve repair, while pyroptosis inducers (e.g., axitinib) combat chemoresistant tumors. We then identify critical knowledge gaps and emerging frontiers. The roles of most gasdermins (GSDMA, GSDMB, GSDMC) in PN are unknown. We explore the emerging concept of PANoptosis (crosstalk among pyroptosis, apoptosis, and necroptosis) as a novel conceptual framework for PNS pathologies, where shared molecular hubs may amplify neuroinflammation. Furthermore, despite promising strategies like combination therapy and drug repurposing, a significant translational gap exists, with no current clinical trials specifically targeting pyroptosis for PN.

**Conclusion and Perspective:**

Targeting pyroptosis is a novel therapeutic avenue for PN. This review synthesizes current mechanistic understanding, evaluates preclinical therapeutic strategies, and delineates crucial future directions, including elucidating gasdermin diversity, validating PANoptosis, and bridging the translational divide, thereby accelerating their application for patients suffering from PN.

## Graphical abstract

Pyroptosis is a key inflammatory cell death pathway in peripheral neuropathy, with opposing roles: it perpetuates neuropathic pain but can be induced to kill neuroblastoma cells. This review consolidates mechanistic understanding and therapeutic strategies, arguing for a shift from disease‐centric to target‐centric treatment approaches to improve patient outcomes.

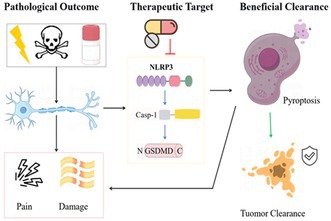

## 1 Introduction

Peripheral neuropathy (PN) encompasses a range of conditions characterized by damage or dysfunction of the peripheral nerves. Diabetes, injury, infection, autoimmune disease, hereditary factors, toxin exposure, tumors, and other unknown reasons cause PN. Its pathological alterations include reduced axonal transport, axonal degeneration, Schwann cell damage, segmental demyelination, and total Wallerian degeneration. Diabetic peripheral neuropathy (DPN), trigeminal neuralgia (TN), postherpetic neuralgia (PHN), neuroblastoma, and peripheral nerve injury (PNI) caused by sciatic nerve crush/chronic constriction injury (CCI) represent common and distinct forms of PN. Patients with peripheral neuropathy must endure various dysfunctions caused by sensory and motor problems, as well as neuropathic pain and related lesions.

While single‐cell sequencing has revolutionized our understanding of cellular drivers behind sensory‐motor deficits and neuropathic pain [1], the involvement of inflammatory programmed cell death (PCD) pathways, particularly pyroptosis, remains underexplored in peripheral neuropathy. Pyroptosis, apoptosis, autophagy, necroptosis, and ferroptosis are all forms of programmed cell death, and many communications and interactions among them [2, 3, 4, 5]. Pyroptosis, an inflammatory form of PCD, relies heavily on plasma membrane (PM) pores generated by the gasdermin (GSDM) family. It is characterized by cell enlargement, membrane rupture, and the release of intracellular contents [6]. Generally, it is classified as an inflammasome‐dependent pyroptosis pathway, which contains the canonical pathway with an inflammasome generated by caspase‐1 [7] and the noncanonical pathway with an inflammasome formed by caspase‐4/5/11 [8]. Moreover, there are also inflammasome‐independent pathways, which are activated by caspase‐3/8, Granzyme A, Granzyme B, streptococcal pyrogenic exotoxin B (SpeB) virulence factor, and Neutrophil elastase, Cathepsin G [9, 10, 11, 12, 13]. Increasing evidence has confirmed the important roles of pyroptosis in various diseases, and it is being targeted for preclinical treatment [14, 15, 16, 17, 18]. However, there are just a few findings on the peripheral nervous system (PNS). Therefore, we thoroughly searched PubMed, Scopus, Web of Science, and Google Scholar to demonstrate the potential involvement of pyroptosis in PNS and emphasize the therapeutic implications for peripheral neuropathy. This review bridges molecular insights to translational innovation by addressing critical gaps in the current neuroimmune therapeutics approaches. It advocates for mechanism‐driven clinical trials that leverage pyroptosis modulation to develop next‐generation therapies.

## 2 Methods

Although the concept of “pyroptosis” was first proposed in 2001 [19], the aberrant cell death process was originally identified in 1986 [20]. Thus, we searched publications from January 1, 1986, to November 30, 2025.

### 2.1 Search Strategy

A systematic literature search was conducted to identify all relevant studies investigating the role of pyroptosis in PN. The search period spanned from January 1, 1986, to November 30, 2025. Four electronic databases were searched: PubMed, Scopus, Web of Science Core Collection, and Google Scholar. To ensure comprehensiveness, the search strategy was built around two core conceptual blocks: (1) Peripheral Neuropathy and its subtypes, and (2) Pyroptosis and its molecular mechanisms.

The search employed a combination of controlled vocabulary (e.g., Medical Subject Headings [MeSH] in PubMed) and free‐text keywords in the title, abstract, and author keyword fields. The specific terms for each conceptual block are detailed below:

Block 1 (Peripheral Neuropathy): Terms included, but were not limited to: “peripheral neuropathy,” “peripheral nervous system diseases,” “diabetic neuropathies,” “neuropathic pain,” “sciatic nerve injury,” “chronic constriction injury,” “trigeminal neuralgia,” “postherpetic neuralgia,” “neuroblastoma,” and “schwannoma”.

Block 2 (Pyroptosis): Terms included, but were not limited to: “pyroptosis,” “gasdermin” (including GSDMA, GSDMB, GSDMC, GSDMD, GSDME), “inflammasome” (including NLRP3, AIM2, ASC), “caspase‐1,” “caspase‐4/5/11,” “caspase‐3,” “caspase‐8,” “IL‐1β,” and “IL‐18”.

The full reproducible search syntax for each database is provided in Appendix S1.

### 2.2 Study Selection and Eligibility Criteria

All retrieved records were imported into EndNote 2025 reference management software. Duplicates were removed using the software's automated function, followed by manual verification.

The study selection process adhered to the Preferred Reporting Items for Systematic Reviews and Meta‐Analyses (PRISMA) guidelines. Titles and abstracts of unique records were screened by two independent reviewers against predefined eligibility criteria. Any discrepancies were resolved through discussion or by consultation with a third reviewer.

The eligibility criteria were as follows: (1) Population/Model: In vivo or in vitro models relevant to peripheral nervous system disorders, or human samples from PN conditions; (2) Intervention/Exposure: Studies that experimentally modulated or investigated pyroptosis pathways (e.g., using genetic knockout, pharmacological inhibitors/activators, or measuring pyroptosis markers); (3) Comparator: Appropriate control groups (e.g., sham‐operated, vehicle‐treated, wild‐type); (4) Outcome: Reports on molecular, cellular, or behavioral outcomes related to pyroptosis in the context of PN pathogenesis, pain, or nerve regeneration; (5) Study Design: To ensure our synthesis was based purely on primary data and to avoid repeating the conclusions of previous reviews, only original, full‐length research articles were included to ensure synthesis was based on primary data. Review articles, meta‐analyses, conference abstracts, editorials, and non‐English publications were excluded at the full‐text screening stage.

After the initial screen, the full texts of potentially eligible articles were obtained and assessed against the same criteria. The entire selection process, documenting the number of records at each stage, is presented in a PRISMA flow diagram (Figure S1).

### 2.3 Data Extraction and Synthesis

Data from the included studies were extracted into a standardized form. The extracted information included: (1) study characteristics (authors, year, model/cell type); (2) pyroptosis‐related interventions and pathways investigated; (3) key molecular findings (e.g., changes in inflammasome components, gasdermin cleavage, cytokine release); (4) phenotypic outcomes (e.g., pain behavior, nerve conduction, histopathology, cell viability); and (5) main conclusions regarding the role of pyroptosis. Given the expected heterogeneity in models, interventions, and outcomes, a narrative synthesis was performed to summarize and critically discuss the findings thematically, rather than as a quantitative meta‐analysis.

## 3 Pyroptosis: Mechanisms and Pathways in Peripheral Neuropathy

**FIGURE 1:**
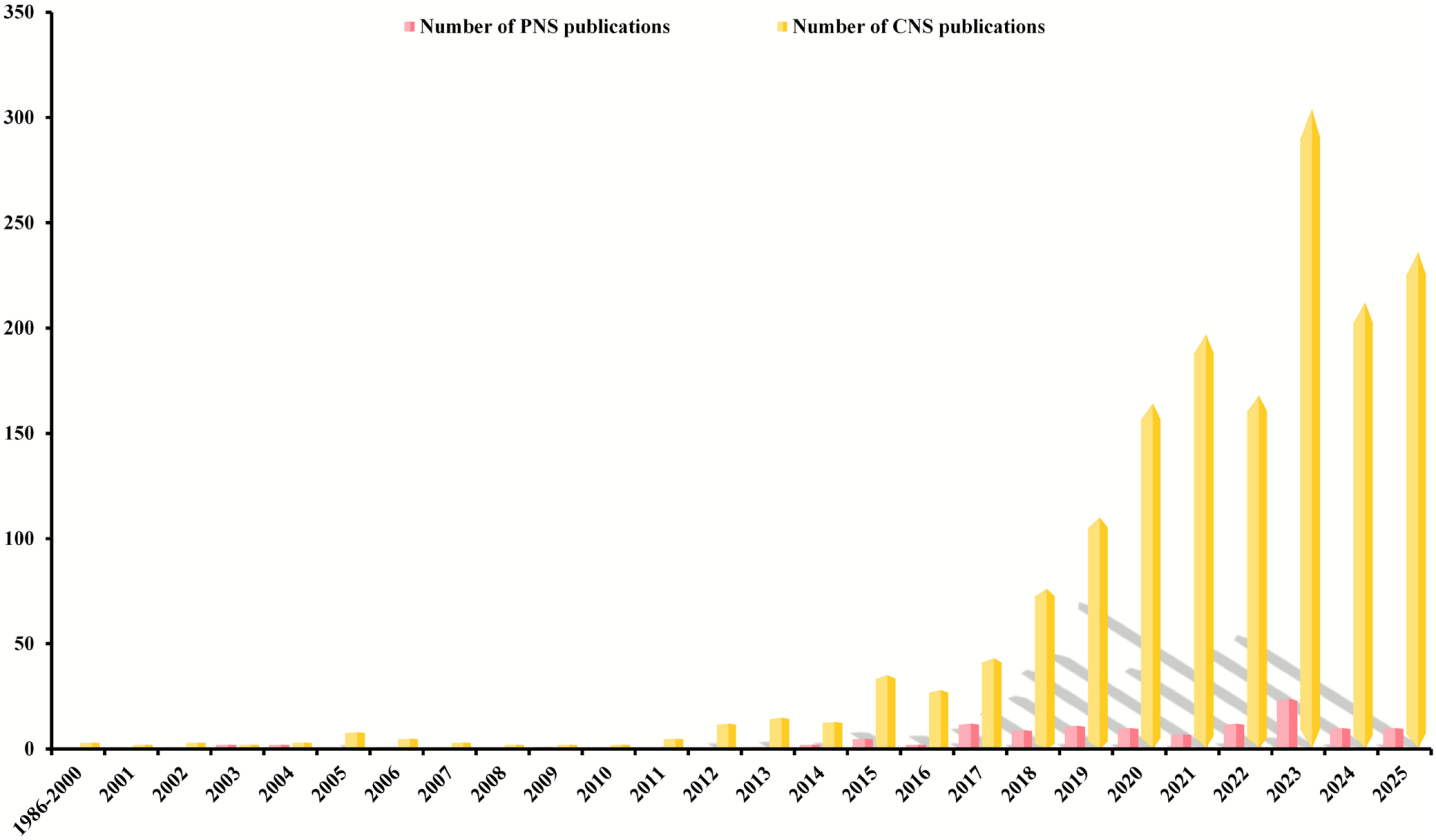
Temporal trends in pyroptosis‐related research within the nervous system: A bibliometric analysis of PubMed‐indexed literature (1986–2024). Data depict the annual publication output of studies investigating pyroptosis mechanisms in various diseases, including those of the central nervous system (CNS) and peripheral nervous system (PNS). The temporal scope (January 1, 1986, to August 31, 2024) was selected to capture the full trajectory of pyroptosis research since its initial mechanistic characterization.

After searching and classifying, more than 10,000 publications demonstrated the role of pyroptosis in various diseases (Table 1). There were only 10 pieces of literature on pyroptosis before 2001, three of which were related to the central nervous system (CNS), with no report on PNS (Figure 1). Pyroptosis continued to receive little attention from researchers for the following 10 years. The number of articles about pyroptosis in the nervous system has increased gradually since 2011; however, fewer than 5% of these studies have explored PNS‐specific mechanisms.

**TABLE 1:** PubMed searches for pyroptosis and various diseases.

Year	Number of all publications	Number of CNS publications	Number of PNS publications
1986–2000	10	3	0
2001	10	2	0
2002	12	3	0
2003	13	2	2
2004	21	3	2
2005	30	8	0
2006	31	5	1
2007	48	3	0
2008	59	2	0
2009	57	2	1
2010	83	2	0
2011	146	5	0
2012	141	12	0
2013	216	15	1
2014	241	13	2
2015	332	35	5
2016	334	28	2
2017	382	43	12
2018	564	76	9
2019	763	110	11
2020	975	164	10
2021	1439	197	7
2022	1175	168	13
2023	2321	304	25
2024	3033	212	10
2025	3361	236	10

Abbreviations: CNS, central nervous system; PNS, peripheral nervous system.

### 3.1 Caspases: Mediate Pyroptotic Pathways in Neurological Pathologies

Humans have caspase‐1, ‐2, ‐3, ‐4, ‐5, ‐6, ‐7, ‐8, ‐9, ‐10, and ‐14, whereas mammals have caspase‐1, ‐2, ‐4, ‐5, ‐8, ‐9, ‐10, ‐11, and ‐12. The inactive form of procaspases is activated when the signaling pathway is triggered, splitting into large and small subunits to form the enzyme complex, which induces a series of events that ultimately result in inflammation or cell death. Caspase‐1, ‐4, ‐5, ‐11, and caspase‐3, ‐8, which trigger the activation of pyroptotic cell death (pyroptosis), are the subject of this discussion. However, emerging evidence suggests that caspase convergence or cross‐regulation is common in pyroptosis.

#### 3.1.1 Canonical Caspase‐1‐Dependent Pyroptosis

Caspase‐1, also known as interleukin‐1 (IL‐1)‐converting enzyme (ICE), was first found in 1989 and was thought to be the only caspase involved in pyroptosis over a prolonged duration [21, 22]. The inflammasome is the primary cause of caspase‐1, which initiates the classical pyroptosis pathway. In addition to cleaving the N‐terminal sequence of gasdermin D (GSDMD) to connect to the membrane and create membrane pores, caspase‐1 also cleaves and activates inflammatory proteins, including IL‐18 and IL‐1β, which causes pyroptosis [23, 24]. Intriguingly, while caspase‐1 triggers GSDMD‐mediated pyroptosis in various peripheral neuropathies [25, 26, 27, 28, 29, 30, 31], caspase‐1 instead activates gasdermin A (GSDMA) or gasdermin E (GSDME) in the CNS disorders [32, 33].

#### 3.1.2 Noncanonical Caspase‐4/5/11 Activation Pathways

Human caspase‐4, ‐5, and mouse caspase‐11 can be directly activated by interaction with lipopolysaccharide (LPS) of Gram‐negative bacteria, and subsequently cleave GSDMD or indirectly activate caspase‐1, thus producing the nonclassical pyroptosis pathway [34, 35, 36]. Caspase‐4/5/11‐dependent neuronal pyroptosis has also been observed in the colonic myenteric neurons of overweight and obese humans and Western diet‐fed (WD‐fed) mice [35], as well as in the mice given complete Freund's adjuvant (CFA) injections [36]. Emerging studies link this pathway to obesity‐associated neuropathy, exposing a targetable metabolic‐inflammatory nexus in PNS pathogenesis.

#### 3.1.3 Caspase‐3/8: Molecular Interactors in PANoptosis

Caspase‐8 was originally demonstrated to mediate apoptosis and contribute to necroptosis. Later, it was discovered that Caspase‐8 could activate GSDMD, gasdermin C (GSDMC), and GSDME to induce pyroptosis [37, 38, 39, 40]. As such, it is crucial to the interaction among “PANoptosis,” which primarily consists of the three types of programmed cell death: necroptosis, apoptosis, and pyroptosis [41, 42]. While the evidence for PANoptosis in the PNS is still in its infancy, we apply this novel conceptual framework throughout the review to synthesize existing data and propose a hypothesis that the co‐regulation of pyroptosis, apoptosis, and necroptosis may underlie the complex pathophysiology and varied therapeutic responses observed in peripheral neuropathies.

Caspase‐3, while canonical to apoptotic execution, exhibits functional duality in pyroptotic pathways [33]. Interestingly, caspase‐3 acts as a bridge between pyroptosis and apoptosis by coordinating with other caspases. GSDMD‐dependent pyroptosis was facilitated by caspase‐3 and caspase‐7 (downstream of caspase‐1) in microglia [43]. In the spiral ganglion neurons of GSDME mutant (without exon 8) injected mice, caspase‐3 was activated by caspase‐9 and subsequently augmented apoptosis, as well as GSDED‐ and GSDEM‐dependent pyroptosis [44]. Moreover, active caspase‐3 and caspase‐4 cleaved GSDMA to induce pyroptosis under African swine fever virus (ASFV) attack in porcine [45].

Collectively, these data position caspase‐3/8 as central orchestrators of PANoptosis, wherein pyroptotic, apoptotic, and necroptotic machinery coalesce into a synergistic death‐inducing platform.

### 3.2 Inflammasomes: Sensors and Inducers

Within the classical pyroptosis pathway, inflammasomes function as molecular hubs that both detect pathogenic/danger signals and directly initiate the caspase‐1‐dependent pyroptotic pathway—the keystone innate immune response. Each inflammasome has three main components: the adaptor NOD‐like receptor family pyrin domain containing 3 (NLRP3) protein ASC, the effector protein (caspase family), and the sensor (leucine‐rich repeat‐containing proteins [NLRs] family or the absent in melanoma 2‐like receptors [ALRs] family). Distinct inflammasome subtypes orchestrate responses to specific endogenous or exogenous triggers, with NLRP3 dominating in PNS pathologies. Emerging evidence identifies nonredundant roles for NLRP6 and AIM2 inflammasomes in peripheral nerve disorders, activated through divergent upstream sensors yet converging on shared pyroptotic execution.

#### 3.2.1 NLR Family: Structural Diversification and Pathogenic Roles

The NLR family can be divided into three subfamilies: the NLRP subgroup (namely NLRP1, 2, 3, 4, 5, 6, 9, 10) with a pyrin domain (PYD), the NLRC subgroup (namely NLRC4/IPAF, NOD1, NOD2) with a caspase activation and recruitment domain (CARD), and the NLRB/NAIP subgroup (namely NAIP1, 2, 5, 6) with baculovirus inhibitor repeat (BIR) domains [46, 47]. Under normal conditions, all 15 NLR members were expressed in the most common PNS cell types with cell‐type‐specific differences [48]. Most members of the NLR family were activated in peripheral nerve‐injured conditions and could work with others to cause pyroptosis [48, 49, 50, 51]. The increased level of NLRP3 in the Schwann cells of the DPN model [52] and injured sciatic nerve [53] indicated pyroptosis. The elevation of NLRP2 in the dorsal root ganglia (DRG) caused pyroptosis and pain induced by peripheral nerve injury [54]. *NLRP6*‐deficient mice showed delayed recovery of sciatic nerve function following sciatic nerve damage compared to *ASC*‐, *Nlrp3*‐, or *Caspase*‐*1*‐deficient mice, which suggested that NLRP6 has unique nonredundant functions in neural repair mechanisms [48].

#### 3.2.2 ALR Family: Emerging Regulators of Neural Inflammation

ALRs family, also known as the pyrin and HIN domain (PYHIN) family, has PYD and HIN200 domain. IFN‐γ‐inducible protein 16 (IFI16) and absent in melanoma 2 (AIM2) belong to this family. While IFI16 shows no documented pyroptotic activity in neural tissues, AIM2 has been thoroughly investigated in peripheral neuropathies [55, 56, 57, 58].

ASC specks are protein complexes formed when the inflammasome is activated and are thought to be a sensitive and specific potential plasma biomarker in some disorders [59]. The sciatic nerve, Schwann cell, sensory neuron, and motor neuron all presented high ASC expression [48]. Peripheral nerve‐injured mice showed progressive ASC speck accumulation with concomitant increases in both ASC transcript levels and protein expression [51, 53, 60, 61], suggesting its utility as a dynamic marker of inflammasome activity in PN.

### 3.3 Gasdermins: Executors of Pyroptotic Death

The discovery of the Gasdermin (GSDM) family has greatly expanded our understanding of pyroptosis [12, 62, 63, 64]. In humans, the Gasdermin family comprises six members: five pore‐forming proteins (GSDMA–GSDME) and one non‐pyroptotic member (deafness autosomal recessive [DFNB] 59, also known as pejvakin [PJVK]). In mice, GSDMA has three homologs (GSDMA1‐3), GSDMC has four homologs (GSDMC1‐4), while GSDMD, GSDME, and PJVK have one homolog each. Notably, GSDMB is absent. This review focuses on the pyroptosis‐inducing GSDMs (GSDMA–GSDME). Following GSDM cleavage, the peptides of the N‐terminal (NT) active domain in cells typically clump together on the cell membrane and attach to membrane lipids to form pores. These pores upset the cell's osmotic pressure, resulting in swelling, rupture, and release of inflammatory factors such as IL‐1β and IL‐18 [65]. Intriguingly, full‐length GSDME was found to mediate pyroptosis under high UV‐C irradiation, representing a unique activation mechanism [66].

**FIGURE 2:**
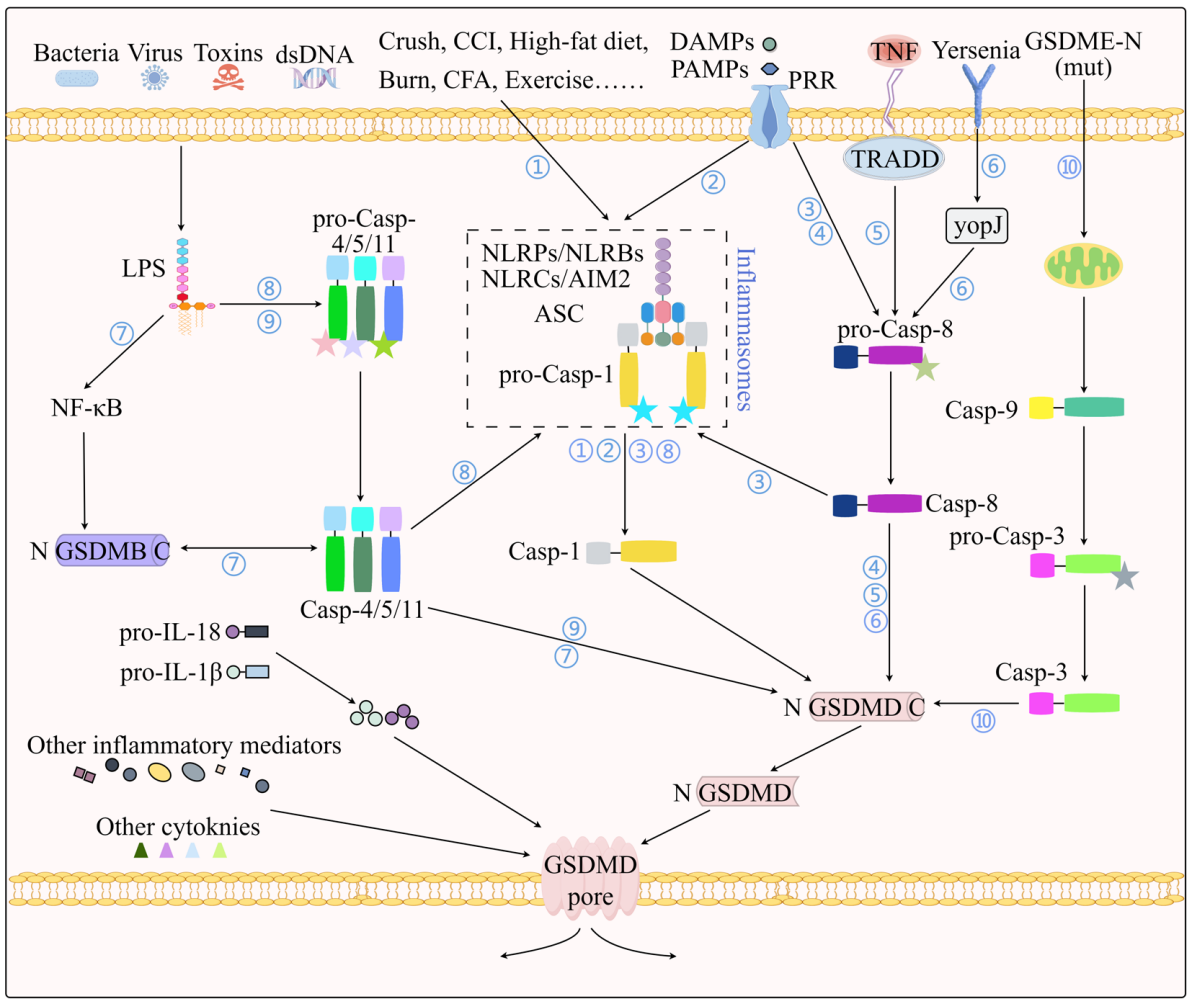
Molecular convergence of GSDMD‐dependent pyroptotic signaling in peripheral neuropathy. This schematic illustrates the principal molecular pathways through which gasdermin D (GSDMD)‐mediated pyroptosis is activated, highlighting its role in the development of peripheral neuropathy. The figure delineates canonical (inflammasome‐dependent) and noncanonical pathways, showing how diverse stimuli converge on GSDMD cleavage. Proteolytic release of the GSDMD N‐terminal fragment (GSDMD‐NT) leads to oligomerization and plasma membrane pore formation, resulting in lytic cell death and release of pro‐inflammatory cytokines interleukin‐1β (IL‐1β) and interleukin‐18 (IL‐18). The numerically annotated pathways (1–10) represent distinct triggers and their primary signaling routes to GSDMD activation. Pathways with direct evidence in PN: ① Initial insults, including mechanical injury (e.g., CCI, crush), metabolic stress (e.g., high‐fat diet), inflammatory challenges (e.g., burn, CFA), and exercise, are established triggers of inflammasome‐mediated pyroptosis in PN models. ② Damage‐associated molecular patterns (DAMPs) and pathogen‐associated molecular patterns (PAMPs) engage pattern recognition receptors (NLRP3, NLRBs, NLRCs, AIM2), leading to inflammasome assembly and caspase‐1 activation. Experimentally validated in PN models. Pathways with emerging or indirect evidence in PNS contexts: ③ Caspase‐8‐mediated inflammasome priming: A subset of DAMPs/PAMPs can activate caspase‐8, which in turn can promote the activation of the canonical inflammasome pathway. PNS‐specific mechanisms need further investigation. ④ Direct caspase‐8 cleavage of GSDMD: Activated caspase‐8 can directly cleave and activate GSDMD. Its significance in PN remains exploratory, but it is a mechanism of interest. Pathways established in other systems with potential relevance to PN: ⑤ TNF signaling: TNF leads to caspase‐8 activation and subsequent GSDMD cleavage. This pathway's role in PNS is currently supported by indirect evidence from Alzheimer's disease (AD) models and requires further direct validation in peripheral nerve‐specific contexts. ⑥ Yersinia infection: Infection by *Yersinia* bacteria directly activates caspase‐8, triggering GSDMD‐dependent pyroptosis. This pathway's role in PNS is currently supported by indirect evidence from macrophage models and requires further direct validation in neuronal contexts. ⑦ GSDMB‐dependent noncanonical route: Cytosolic LPS can trigger the formation of GSDMB pores, which facilitate the activation of human caspase‐4/5 or murine caspase‐11. The role of this pathway in the PNS is currently speculative, with supporting evidence from blood–brain barrier studies (LPS‐caspase‐4/11 axis) and limited direct neuronal data. ⑧ Inflammasome‐augmented noncanonical route: Cytosolic LPS‐activated caspase‐4/5/11 can engage inflammasome components to amplify GSDMD cleavage. The crosstalk between caspase‐4/5/11 and inflammasomes is experimentally validated in blood–brain barrier models, while its role in PNS neuroinflammation remains to be fully characterized. ⑨ Direct noncanonical route: Cytosolic LPS directly binds and activates human caspase‐4/5 or murine caspase‐11, leading to GSDMD cleavage, potentially relevant in PNS infections; ⑩ Mutant GSDME Trigger: A GSDME‐N‐terminal mutant can initiate an intrinsic apoptotic cascade involving caspase‐9 and caspase‐3, which ultimately converges on GSDMD activation. Based on in vitro models, the physiological relevance of intact PNS requires confirmation. This figure integrates pathways with varying levels of experimental support in the peripheral nervous system. Pathways 1 and 2 are supported by direct evidence from PN models. Pathways 3–9 are primarily defined in nonneuronal contexts (e.g., immunology, epithelial biology), and their specific roles in PN remain hypothetical and crucial future research directions. Pathway 10 serves as a proof‐of‐concept for cell death crosstalk. ASC, apoptosis‐associated speck‐like protein containing a CARD; CCI, chronic constriction injury; CFA, complete Freund's adjuvant; DAMP, damage‐associated molecular pattern; GSDMD, gasdermin D; GSDMD‐NT, GSDMD N‐terminal fragment; IL, interleukin; LPS, lipopolysaccharide; NF‐κB, nuclear factor kappa‐light‐chain‐enhancer of activated B cells; PAMP, pathogen‐associated molecular pattern; Pro‐Casp, procaspase; TNF, tumor necrosis factor.

GSDMD serves as the primary substrate for all inflammatory caspases (caspase‐1, 4/5/11, ‐3, 8) that participate in the pyroptosis process (Figure 2). Other GSDMs exhibit caspase specificity: GSDMA is cleaved by caspase‐1, ‐3, and ‐4; GSDMB is cleaved by caspase‐7 to form GSDMB pore and interact with caspase‐4 to trigger GSDMD pore; GSDMC is cleaved by caspase‐8 to form GSDMC pore; and GSDME is cleaved by caspase‐1 and ‐3 to form GSDME pore [6, 62, 67, 68, 69, 70]. Beyond caspases, bacterial proteases (e.g., SpeB cleaving GSDMA) and granzymes (granzyme A [GzmA]/granzyme B [GzmB] cleaving GSDMB/GSDME, respectively) activate GSDMs, highlighting their role in host–pathogen interactions [11, 12, 71].

#### 3.3.1 GSDME

**FIGURE 3:**
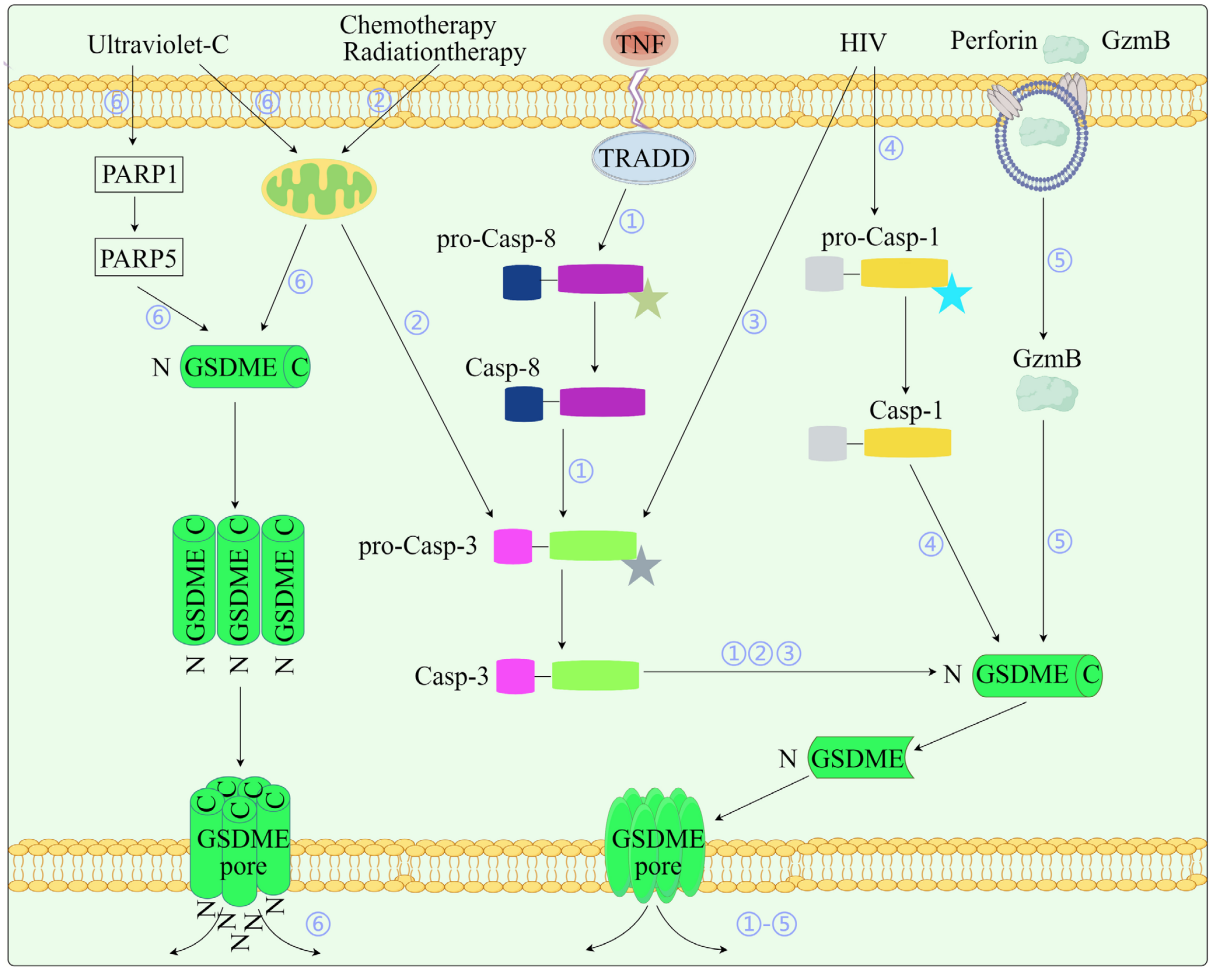
Mechanistic diversity of GSDME‐dependent pyroptotic signaling in therapeutic and pathological contexts. This schematic delineates stimulus‐specific activation pathways of gasdermin E (GSDME)‐mediated pyroptosis, highlighting its dual role as both a therapeutic effector in cancer treatment and a pathological mediator in neurological disorders. The numerically annotated pathways (1–6) represent distinct molecular routes to GSDME activation: ① TNF‐mediated neuronal pyroptosis in Alzheimer's Disease (AD): Tumor necrosis factor (TNF) signaling activates caspase‐8, which subsequently triggers the caspase‐3/GSDME pathway, contributing to neuronal pyroptosis. Supported by studies in AD models. ② Chemotherapy‐induced cytotoxicity: Chemotherapeutic drugs (e.g., cisplatin, etoposide) activate caspase‐3 through the intrinsic apoptotic pathway, leading to GSDME cleavage and pyroptosis in neuroblastoma and other cancer cells. Well‐established in neuroblastoma cell lines and chemotherapy‐induced peripheral neuropathy models. ③ HIV‐associated neuropathology: HIV infection may contribute to peripheral nervous system pathology through caspase‐3/GSDME‐mediated pyroptosis. Primarily demonstrated in HIV‐infected immune cells (e.g., CD4^+^ T cells); direct evidence in neurons remains limited and requires further validation. ④ CNS HIV infection and pyroptosis: HIV infection in the CNS triggers caspase‐1 activation, leading to GSDME cleavage and neuronal pyroptosis. Evidence for this mechanism is derived from CNS studies, and its relevance to the PNS has not been established. ⑤ Granzyme B‐mediated neuronal death: Granzyme B (GzmB) directly cleaves the N‐terminal domain of GSDME to induce neuronal pyroptosis. GzmB release correlates with neuronal death in AD models; the role in peripheral neuropathy remains unexplored. ⑥ UVC radiation‐induced pyroptosis: High‐dose ultraviolet C (UVC) radiation induces pyroptosis through caspase‐independent oligomerization of full‐length GSDME. Demonstrated in epithelial and skin models; neurological relevance unknown. Among the GSDME‐dependent pathways illustrated, chemotherapy‐induced cytotoxicity (Pathway 2) currently has the most robust direct evidence specifically in the context of peripheral neuropathy. In contrast, the other pathways presented are primarily supported by evidence derived from CNS, immunology, oncology, and other non‐neural systems. Their potential roles in peripheral nerve pathologies remain to be fully elucidated and warrant substantial future investigation. CNS, central nervous system; GSDME, gasdermin E; GzmB, granzyme B; HIV, human immunodeficiency virus; TNF, tumor necrosis factor; UVC, ultraviolet C.

GSDMD is the most extensively researched GSDM, and GSDMD‐mediated pyroptosis is quite common in the PNS [40, 72, 73, 74, 75]. Nevertheless, the GSDME‐dependent pyroptosis pathway is an option for cells with minimal or no GSDMD expression [33, 76, 77] (Figure 3). Originally studied in oncology as DFNA5, GSDME now emerges as a regulator of organ injury, with normal cellular responses having been reported [10, 78, 79, 80]. GSDME can be preferentially cleaved by caspase‐3 and switch apoptosis to pyroptosis via NT pore formation [10, 81]. Interestingly, pyroptosis and apoptosis could be triggered by a GSDME mutant that lacks exon 8 (350 bp) in spiral ganglion neurons, suggesting isoform‐specific functions [44]. By causing mitochondrial damage in axons, GSDME activation could hasten the progression of frontotemporal dementia (FTD) and amyotrophic lateral sclerosis (ALS) [82]. Neuropathic pain is widespread in human immunodeficiency virus (HIV) infected patients [83]. HIV infection in the brain triggers caspase‐1, which in turn triggers caspase‐3 to cleave GSDME and cause neuronal pyroptosis in HIV‐associated neurocognitive disorder (HAND) [33]. While acrylamide (ACR)‐induced SH‐SY5Y pyroptosis involves both caspase‐3/GSDME and caspase‐1/GSDMD pathways [84], herpes simplex virus (HSV) type‐2 triggers the caspase‐2/tBID/caspase‐3/GSDME pathway in the absence of caspase‐1/GSDMD expression [85]. Nonetheless, only a few studies that discovered SH‐SY5Y cells treated with cisplatin (DDP) or etoposide (VP16) [86] and LPS (to imitate postsurgical neuroinflammation) [87] consistently implicate the caspase‐3/GSDME pathway as functionally linked to pyroptosis. There is still much to learn about whether the caspase‐3/GSDME pathway is more widely engaged in peripheral neuronal cell death, as these contradictory findings could be caused by various stressors to the cells.

#### 3.3.2 GSDMA

**FIGURE 4:**
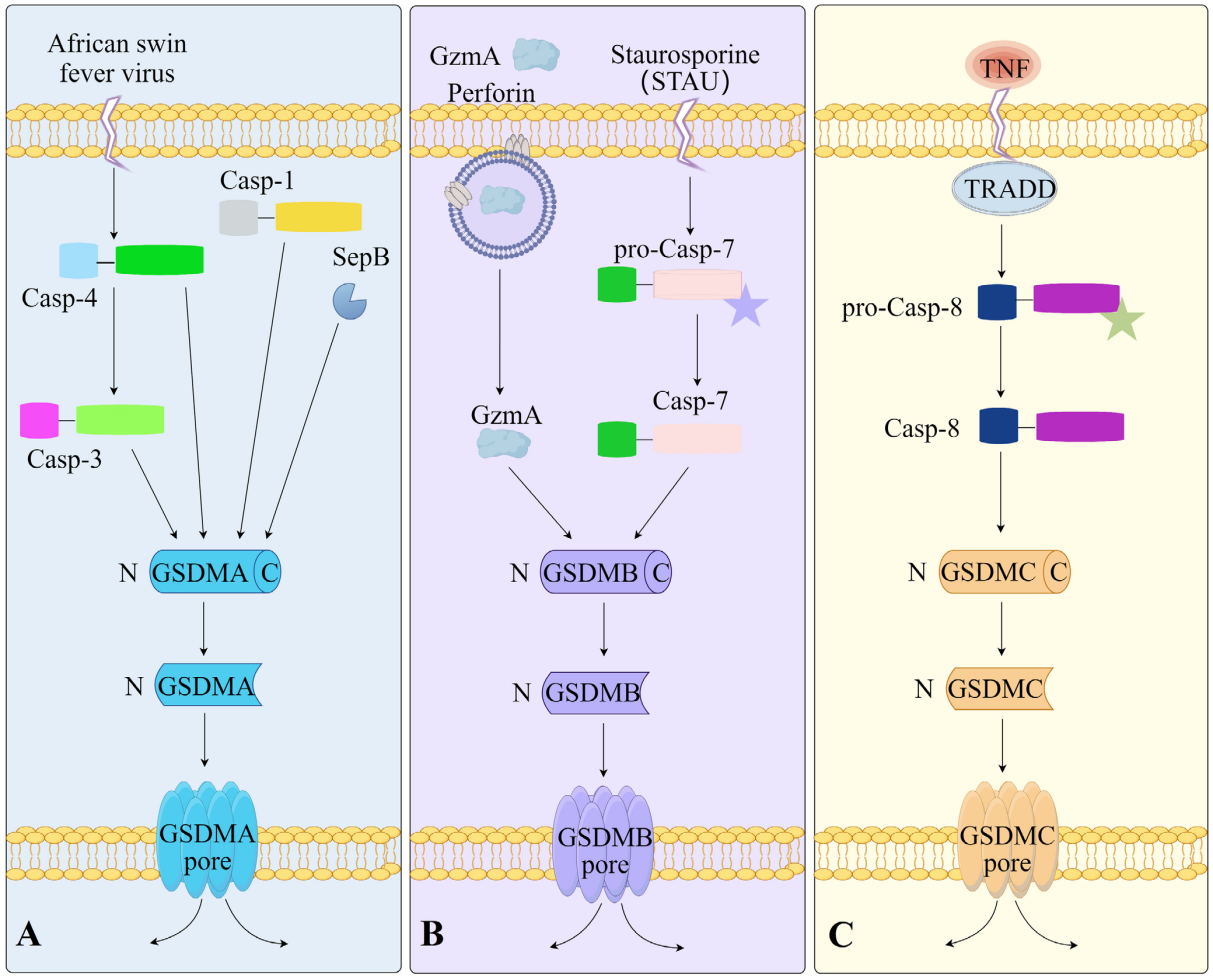
Diverse caspase‐dependent pathways of gasdermin A, B, and C‐mediated pyroptosis in disease pathogenesis. (A) Pathogen‐activated GSDMA‐dependent pyroptotic pathways. This schematic delineates the molecular interplay between caspase activation and GSDMA‐driven pyroptosis, highlighting pathogen‐specific triggers and effector mechanisms. Caspase‐1, streptococcal pyrogenic exotoxin B (SpeB), caspase‐3, and caspase‐4 can induce GSDMA‐dependent pyroptosis. The activation pathways shown for GSDMA are primarily defined in nonneuronal systems. SpeB‐mediated cleavage is established in streptococcal infection models, while caspase‐1 and caspase‐3/4‐mediated cleavage are documented in porcine and avian systems, respectively. The expression and functional role of GSDMA in the peripheral nervous system remain completely unexplored. (B) Granzyme A and caspase‐7‐dependent activation of GSDMB‐driven pyroptosis. This schematic illustrates the molecular mechanisms governing GSDMB‐mediated pyroptosis, highlighting its dual activation by cytotoxic immune effectors and apoptotic stress signals. Key pathways include immune cell‐mediated cytotoxicity (GzmA/Perforin axis) and apoptosis‐to‐pyroptosis switch (caspase‐7‐dependent pathway). GSDMB research is predominantly focused on epithelial cells and immune regulation. While GSDMB polymorphisms are associated with multiple sclerosis susceptibility, direct evidence of GSDMB‐mediated pyroptosis in peripheral nerve cells or neuropathies is currently lacking. These pathways represent hypothetical mechanisms that may be relevant to immune‐mediated neuropathies. (C) TNF‐α/caspase‐8 axis‐driven GSDMC‐dependent pyroptosis. This schematic illustrates the molecular cascade from TNF signaling to GSDMC‐mediated pyroptosis, a key mechanism driving chronic inflammation and therapy‐resistant malignancies. It is well‐documented in cancer and inflammatory diseases, but its role in peripheral neuropathy remains unvalidated. While GSDMC polymorphisms are associated with radiculopathy, functional studies in neural tissues are lacking. GSDM, gasdermin; GzmA, granzyme A; SpeB, streptococcal pyrogenic exotoxin B; TNF‐α, tumor necrosis factor‐alpha.

GSDMA remains understudied in neurology but displays dual sensor‐effector functions (Figure 4A). Upon detection of cytosolic SpeB, GSDMA undergoes SpeB‐mediated cleavage, forming pores that facilitate further SpeB influx, thereby establishing a positive feedback loop that amplifies bacterial virulence [12]. Notably, evolutionary divergence in the cleavage and activation of GSDMA has been observed across species: in humans and mice, SpeB represents the sole pathogen‐encoded protease known to cleave and activate GSDMA. In contrast, during African swine fever virus (ASFV) infection in pigs, caspase‐3 and caspase‐4 mediate GSDMA cleavage [45]. Furthermore, in birds, amphibians, and reptiles, ancestral GSDMA is cleaved and activated by caspase‐1 [32]. Clinically, elevated GSDMA expression is correlated with resistance to anti‐PD‐L1 therapy in glioblastoma, positioning its potential as an immunotherapeutic target [88]. A deeper exploration of GSDMA's activation mechanisms and its functional roles within the PNS constitutes a critical and promising research direction.

#### 3.3.3 GSDMB

GSDMB is a controversial issue because it orchestrates critical biological functions via pyroptosis either in a dependent or independent way in humans [89, 90, 91] (Figure 4B). Its 10 variations exhibit divergent protease interactions: GzmA‐cleaved GSDMB induces pyroptotic cell death in cancer cells [11], whereas neutrophil elastase (ELANE) cleaved GSDMB lacks pyroptotic activity [92]. Unsurprisingly, contradictory reports describe GSDMB's interplay with GSDMD—while some insist it promotes the caspase‐4/GSDMD noncanonical pyroptosis in THP1 cells, others observe no effect [70, 93]. Furthermore, according to Xu et al., the caspase‐7/GSDMB axis regulates the balance between apoptosis and caspase‐4/GSDMD noncanonical pyroptosis. GSDMB binds caspase‐4 to induce pyroptosis, which is inhibited upon LPS‐induced caspase‐7 activation and subsequent cleavage of GSDMB [69]. Clinically, reduced *GSDMB* mRNA affects multiple sclerosis (MS) exacerbations [94, 95]; yet its expression and pathophysiological roles in PNS remain enigmatic.

#### 3.3.4 GSDMC

GSDMC, also known as melanoma‐derived leucine zipper‐containing extranuclear factor (MLZE) [96], is the least characterized GSDM, though emerging roles in tumors, intestinal homeostasis, and gastrointestinal microbe‐sensing skin metabolism highlight its versatility [9, 97, 98, 99] (Figure 4C). Interestingly, in GSDMC‐mediated type 2 immunity, the GSDMC N‐terminal is sufficient to form the pore to facilitate the secretion of other molecules and IL‐33, independent of pyroptosis [98, 100]. According to a single article on the investigation of GSDMC in the nervous system, a genome‐wide association study (GWAS) suggested that CCDC26/GSDMC rs7833174 in susceptibility to low back pain with radiculopathy [101]. Nevertheless, the physiological functions and mechanistic underpinnings of GSDMC within the nervous system remain largely unexplored and merit further investigation.

### 3.4 IL1β and IL18

Following GSDM cleavage, GSDM‐N undergoes oligomerization to form pores, thereby inducing pyroptosis and subsequent release of inflammatory cytokines, including IL‐1β and IL‐18. Elevated expression of IL‐1β and IL‐18 serves as biochemical evidence for inflammasome activation. Notably, coordinated upregulation of IL‐1β, IL‐18, and NLRP3 has been observed in the neurons or Schwann cells of nerve‐injured mice [52, 53, 102]. From a pharmacological perspective, IL‐1β and IL‐18 exhibit dual regulatory functions: while demonstrating antiallodynic and antihyperalgesic effects through neuromodulatory pathways, they simultaneously promote inflammatory cascades contributing to neuropathic pain pathogenesis [103, 104, 105, 106, 107]. This functional dichotomy positions them as promising therapeutic targets for treating PN. Next Generation Sequencing (NGS) RNA sequencing data, combined with experimental validation, has identified IL‐18 as a critical node in peripheral nerve regeneration networks, functioning synergistically with IL‐10, interferon‐gamma (IFN‐γ), and programmed cell death protein 1 (PDCD1) to coordinate peripheral nerve repair [108]. Furthermore, emerging evidence implicates IL‐18 in the pathophysiology of autoimmune demyelinating disease of PNS, suggesting its potential as a therapeutic target for these conditions [109, 110].

## 4 Therapeutic Targeting of Pyroptosis in Peripheral Neuropathy

The elucidation of pyroptosis mechanisms in peripheral neuropathy (PN) has identified multiple druggable targets across the signaling cascade. This knowledge enables a shift from symptom‐ or disease‐centric treatment to a mechanism‐driven strategy, where interventions are designed based on their specific action within the pyroptotic pathway. Here, we synthesize preclinical evidence by reorganizing it according to the molecular logic of intervention: from upstream triggers and inflammasome assembly, through executioner caspase activation and gasdermin pore formation, to context‐dependent therapeutic applications.

A key consideration is the context‐dependent duality of pyroptosis: it acts as a destructive driver of neuroinflammation and pain in most PN forms but exerts beneficial tumoricidal effects in neuroblastoma. This dichotomy guides therapeutic design‐inhibition is prioritized for neuropathic pain relief and nerve repair, while strategic induction is the goal in oncology. The following subsections detail this targeted pharmacopeia, concluding with a discussion of advanced modalities and the translational roadmap for clinical implementation.

### 4.1 Targeting Upstream Regulators and Inflammasome Activation

Inhibiting the initiation of the pyroptotic cascade by targeting upstream signaling hubs represents the most extensively explored therapeutic strategy in PN. The therapeutic relevance of core targets is underscored by their efficacy across multiple etiologically distinct neuropathies.

The purinergic receptor P2X7 (P2X7R)/NLRP3 inflammasome axis has emerged as a master regulatory node implicated across diverse neuropathies [111, 112]. Diabetic peripheral neuropathy (DPN), characterized by progressive nerve damage, sensory deficits, and neuropathic pain [113, 114]. In DPN, the expressions of P2X7R and thioredoxin‐interacting protein (TXNIP) are increased in high glucose‐treated Schwann cells and sciatic nerves, correlating with NLRP3 inflammasome activation and IL‐1β/IL‐18 maturation [52, 115, 116, 117]. Pharmacological blockade of this axis using P2X7R antagonists like Brilliant Blue G (BBG) [31, 118] or compounds such as loganin (the iridoid glycoside isolated from the fruit *Cornus officinalis* ) [52], Tangzu granule (TZG, derived from famous traditional Chinese medicine decoctions) [115], or *N*‐tert‐Butyl‐α‐phenylnitrone (PBN, ROS scavenger) [119] reverses these molecular changes while improving nerve conduction velocity and sciatic neuropathy, rational structural changes. This axis's critical role is not limited to DPN: in trigeminal neuralgia (TN), BBG significantly reduces the neuroinflammatory response in trigeminal ganglia (TGs) [31, 118], and combined use of BBG and the NLRP3 inhibitor MCC950 prevents mechanical hyperalgesia in the sumatriptan (SUMA)‐induced medication overuse headache (MOH) paradigm [120]. Similarly, in postherpetic neuralgia (PHN), BBG reduces the pain threshold and prevents pyroptosis and endoplasmic reticulum stress [121]. In models of musculoskeletal pain, the P2X7 inhibitor A740003, alongside the NLRP3 inhibitor MCC950, reduces activity‐induced pain [29], and BBG raises the mechanical withdrawal thresholds (MWTs) [122]. Beyond P2X7R, the P2X4/NLRP3 signaling pathway speeds up the development of diabetic neuropathic pain, reversible by administering dexmedetomidine (Dex, a highly selective agonist of α2‐adrenergic receptors) [123] or imperatorin (IMP, a secondary product of furan coumarin plant) [124]. Direct inhibition by MCC950 is effective in DPN [125, 126], and slows neurodegeneration in TN [127, 128]. CY‐09, another selective NLRP3 inhibitor, also shows efficacy in DPN models [126]. Bay11‐7082, an IKK‐β/NF‐κB pathway inhibitor that suppresses NLRP3 expression, alleviates pain in lumbar disc herniation models [129].

Multiple other upstream pathways converge to regulate NLRP3 activation, offering additional therapeutic entry points. Therapeutic interventions targeting TXNIP‐NLRP3 have remarkable therapeutic effects on treating DPN, including Jinmaitong (JMT, a traditional Chinese substance) [116] and Verapamil (a known inhibitor of calcium channels) [130]. The CXCL12/CXCR4 chemokine axis, upregulated after sciatic nerve injury, promotes NLRP3 inflammasome activation via NF‐κB phosphorylation and TXNIP expression, leading to Schwann cell pyroptosis and demyelination, which can be reversed by the CXCR4 antagonist AMD3100 [131]. Loganin prevents the CXCL12/CXCR4‐NLRP3/ASC/caspase‐1 axis and attenuates paw withdrawal threshold (PWT) and latency (PWL) [27, 132]. The High Mobility Group Box 1 (HMGB1)/TLR4 axis is activated in type 2 diabetic (T2DM) neural tissues, and its inhibition by glycyrrhizin (GLC) improves nociceptive thresholds [133]. In models of chronic constriction injury (CCI), several regulators have been identified: the C‐type lectin domain containing 7A (Clec7a) (regulated by *Levo*‐tetrahydropalmatine [*l‐*THP] in a dose‐dependent manner) [134, 135], histone deacetylase 6 (HDAC6) (inhibited by ACY‐1215) [136], and the Keap1/Nrf‐2/p62 pathway (augmented by carvacrol [CRC] [137] and paeoniflorin [PF]) [138]. Furthermore, TANK‐binding kinase 1 (TBK1) inhibition by Amlexanox (AMX) suppresses microglial pyroptosis in painful diabetic neuropathy (PDN) [139], while Swertiamarin exerts protective effects via NOX/ROS/NLRP3 pathway inhibition [140]. Connexin43 (Cx43) modulation by its mimetic peptide Peptide5 or Pinocembrin (5, 7‐dihydroxy flavanone, a natural flavonoid) also attenuates pyroptosis and mechanical hypersensitivity in CCI [61, 141].

Noncoding RNA and novel physical/chemical modalities offer innovative approaches to upstream modulation. MicroRNA‐based strategies, such as administration of miR‐186 mimics, reduce CFA‐induced neuropathic pain through the NLRP3 signaling pathway in TGs [142]. Percutaneous balloon compression (PBC) alleviates pain in patients [143]. Hydrogen generated via silicon‐based precursors ameliorates neuroinflammation and demyelination in TN by repressing the NLRP3‐caspase‐1‐GSDMD pathway [144]. Botulinum toxin type A (BoNT/A), a neurotoxin derived from the gram‐positive bacteria *Clostridium botulinum* , has been proven to repress DRG neuron pyroptosis during PHN by upregulating cathelicidin antimicrobial peptide (CAMP) [145, 146]. Repetitive transcranial magnetic stimulation (rTMS) combined with acupuncture can relieve pain in PHN patients by inhibiting NLRP3, caspase‐1, and IL‐1β [147].

Negative regulators such as Adenosine Deaminase Acting on RNA 3 (ADAR3) and microRNAs (e.g., miR‐223, miR‐34c) further fine‐tune this process, whose overexpression represses NLRP3‐mediated pyroptosis and pain [148, 149, 150, 151, 152]. Furthermore, several natural products and dietary components have demonstrated therapeutic potential by broadly suppressing NLRP3 inflammasome activation. For instance, anthocyanins, widely distributed in fruits, vegetables, and flowers, in addition to their known neuroprotective effects on CNS disorders [153], have been shown to reduce peripheral sympathetic nerve activity in the high salt‐induced hypertension rat by blocking the NLRP3 inflammasome [154]. Specialized pro‐resolving mediators (SPMs) like resolvins [155] and Maresin 1 (MaR1) also demonstrate good analgesic effects and strong anti‐pyroptotic activity [156, 157].

Beyond the predominant NLRP3 focus, targeting alternative inflammasomes shows promise. Intrathecal injection of aspirin‐triggered‐15‐epi‐lipoxin A4 (ATL) inhibits the activation of NALP1 inflammasome, relieving thermal pain behaviors [158]. An elevated NLRP2 inflammasome and segregated NLRP1 contribute to persistent pain [54, 159], and NLRP6 and caspase‐11 are upregulated in cyclophosphamide (CYP)‐induced nerve‐injured neurons [160]. Genetic ablation of *NLRC4* or *ASC* mitigates carrageenan‐mediated hypersensitivity [60].

In addition to focusing on these established upstream factors, certain medicines may also directly target pyroptosis or indirectly target it through unidentified upstream factors. An important defense against DPN‐induced nerve damage was provided by the injection of curcumin (CUR) [161], glucagon‐like peptide‐1 receptor agonist (GLP‐RA) [162], tauroursodeoxycholic acid (TUDCA, ER stress modulator) [163, 164], vincamine (Vin) [165], and Açaí berry [166].

In summary, a multitude of upstream regulators—from purinergic receptors and chemokine axes to stress‐sensing kinases, transcriptional pathways, and noncoding RNA—orchestrate the activation of the core pyroptotic machinery in PN. Their broad implication across etiologically distinct neuropathies highlights their fundamental role in pathogenesis and solidifies them as prime targets for mechanism‐driven therapeutic intervention.

### 4.2 Targeting Effector Caspases

Direct pharmacological inhibition of the effector caspases that cleave and activate gasdermins constitutes a potent strategy to halt pyroptosis execution. Caspase‐1, as the canonical inflammatory caspase, is a primary target. Its inhibition has been shown to alleviate neuropathic pain across multiple models. The caspase‐1 inhibitor VX‐765 attenuates pyroptosis in a model of chronic constriction‐injured infraorbital nerves, with superior efficacy in combination therapy [167]. Similarly, Ac‐YVAD‐cmk (a caspase‐1 inhibitor) inhibits the downstream pro‐inflammatory cytokines and alleviates pain caused by peripheral nerve injury [168]. Inhibition of caspase‐1 reduces postsurgical sensitization and inflammation [169]. Genetic ablation studies further support this target; *Caspase‐1* ^−/−^ mice showed lower cyclooxygenase (COX)‐2 protein expression and PGE_2_ production [170], and in the sciatic nerve transection and microanastomosis (SNTM) models, *Caspase‐1* ^−/−^ SNTM mice show better recovery of the sciatic function index (SFI) than the wild‐type (WT) controls [72], highlighting the detrimental role of caspase‐1 activity in the repair process. Pharmacological inhibition of caspase‐1 via Ac‐YVAD‐cmk directly increases sciatic nerve regeneration by mitigating pyroptosis [171]. The upstream protease cathepsin B (CatB), involved in pro‐caspase‐1 activation, is another target, and its inhibition by CA‐074Me or genetic deficiency dramatically decreases inflammatory pain [104].

Beyond caspase‐1, targeting caspase‐3 and caspase‐8 is crucial, particularly in the context of noncanonical pyroptosis and the emerging PANoptosis paradigm, where these caspases serve as molecular hubs. Inhibition of these caspases may therefore modulate the complex crosstalk between cell death modalities, offering a strategy to fine‐tune the cellular response to injury; however, this approach requires precise contextual application to avoid disrupting essential homeostatic functions.

### 4.3 Targeting Gasdermin Pore Formation and Execution

Directly inhibiting the final, executioner step of pyroptosis‐gasdermin‐mediated pore formation represents a strategy to block lytic cell death and inflammatory content release regardless of the upstream activating pathway. The most prominent target is GSDMD, the primary executioner protein for inflammasome‐activated pyroptosis. Genetic evidence strongly supports its pivotal role; in the SNTM models, *GSDMD* ^−/−^ mice exhibit superior functional recovery compared to mice deficient in upstream inflammasome components like *NLRP3* ^−/−^, *NLRC4* ^−/−^, and *AIM2* ^−/−^ [72], underscoring its nonredundant, terminal position in the pathogenic cascade. Therapeutic intervention of GSDMD has shown promise. The alcohol‐abuse drug disulfiram (and its metabolite dimethyl fumarate, DMF) has been identified as a direct covalent inhibitor of GSDMD pore formation, protecting against hearing loss by inhibiting pyroptosis in spiral ganglion neurons [44]. In trigeminal neuralgia models, a combinatorial therapy incorporating GSDMD‐specific siRNA alongside a caspase‐1 inhibitor (VX‐765) synergistically suppressed pyroptosis, suggesting that co‐targeting the activator and the executor can enhance therapeutic outcomes [167]. In brachial plexus root avulsion injury, Edaravone (EDA) has been shown to improve motor recovery by inhibiting NLRP3/GSDMD/caspase‐1‐mediated pyroptosis and neuroinflammation [172]. This aligns with broader findings that GSDMD oligomerization is regulated by cellular metabolic pathways, such as the Ragulator‐Rag‐mTORC1 axis, revealing novel immunomodulatory checkpoints that could be exploited for therapeutic gain [173].

Beyond GSDMD, modulation of other gasdermins is emerging, particularly in specific contexts. GSDME, cleaved by caspase‐3, can switch apoptotic signals to pyroptotic outcomes. In neuroblastoma, this pathway is activated by chemotherapy drugs (e.g., cisplatin, etoposide) and targeted agents like axitinib, leading to tumoricidal pyroptosis [10, 86, 174, 175]. Conversely, in certain neurological conditions, GSDME activation can mediate mitochondrial damage in axons and contribute to neurodegeneration [82]. While pharmacological inhibition of GSDME is less developed, its context‐dependent role highlights the potential of therapeutic induction in oncology and the need for precise inhibition in neurotoxic settings. The roles of GSDMA, GSDMB, and GSDMC in peripheral neuropathy remain largely unexplored, representing a significant knowledge gap. However, bioinformatic analyses have identified a pyroptosis‐related gene signature including GSDMD, NLRP3, and NLRC4 that correlates with neuropathic pain severity, validating these executioner molecules as clinically relevant biomarkers and targets [176].

Therefore, direct targeting of gasdermin pore formation, especially GSDMD, offers a powerful downstream intervention point to halt pyroptosis. The efficacy of agents like disulfiram and genetic ablation data underscore the therapeutic value of this approach. The divergent roles of GSDME, as a therapeutic inducer in cancer and a potential pathological mediator in neurons, exemplify the “double‐edged sword” nature of pyroptosis executors and underscore the necessity for mechanism‐ and context‐specific therapeutic strategies.

### 4.4 Exploiting the Dual Role: Therapeutic Induction of Pyroptosis in Oncology

The duality of pyroptosis is most strikingly demonstrated in neuroblastoma, the most common extracranial solid tumor encountered in children [177, 178], where its strategic induction serves as a powerful tumoricidal mechanism. This paradigm exploits the high basal expression or inducibility of specific gasdermins, particularly GSDME, in cancer cells. Pyroptosis exhibits stimulus‐dependent duality in SH‐SY5Y neuroblastoma cells pathogenesis: chemotherapy drugs (topotecan, etoposide [VP16], cisplatin [DDP], CPT‐11, or cisplatin) predominantly activate GSDME‐mediated pyroptosis through caspase‐3 cleavage [10, 86, 175]; pathogen/metabolic stressors (enterovirus 71 [EV71] [57, 179], high cholesterol level [180], Aβ_25–35_ [181], and sodium fluoride [NaF] [182]) trigger GSDMD‐mediated pyroptosis via NLRP3 inflammasome activation; hypoxia‐mimetic agents CoCl_2_ treatment induces dual pyroptotic pathways involving both NLRP3/caspase‐1/GSDMD and caspase‐3/GSDME cascades [183]. Notably, NLRX1 inflammasome‐mediated pyroptosis in Enterovirus A71 (EV‐A71) infected SH‐SY5Y cells could be repressed by miR‐195 via direct NLRX1 binding [184]. While caspase‐3/GSDME‐dependent pyroptosis was observed following axitinib treatment in the mouse neuroblastoma 9464D cells, 975A2 cells, and 9464D implanted NB syngeneic mouse models [174], the NLRP3/caspase‐1/GSDMD pathway was found in the human neuroblastoma cell lines, IMR‐32 and SK‐N‐SH cells, with bisphenol A (BPA) treatment [73]. Intriguingly, a prediction model with NLRP3/CASP3/IL18/GSDMB was developed, and the high expression of these genes in neuroblastoma correlates with improved survival [185]. This stands in distinct contrast to other malignancies, where pyroptosis may promote tumor progression, highlighting the unique therapeutic window in NB. Therefore, for high‐risk neuroblastoma, therapeutic strategies should be designed to augment or induce pyroptosis, a fundamental reversal of the therapeutic goal for neuropathic pain.

### 4.5 Advanced and Cell‐Type‐Specific Therapeutic Modalities

Beyond small molecule inhibitors, advanced therapeutic modalities offer enhanced specificity and novel mechanisms of action. Healthy Schwann cells are essential for regenerating nerve fibers and undergo pyroptosis in response to PNI [171, 186]. Schwannomas are slow‐growing peripheral nerve sheath tumors that can cause extreme pain, deafness, dizziness, sensory/motor dysfunction, and even death [187, 188]. Innovative gene therapies exploiting pyroptosis mechanisms show preclinical efficacy: direct intratumoral (I.T.) injection of HSV or adeno‐associated virus (AAV) amplicon vector that contains the Schwann cell‐specific P0 promoter and pyroptosis factor (e.g., caspase‐1 [ICE], N‐terminal of GSDMD [GSDMDNterm], and ASC) could successfully alleviate Schwannoma‐associated pain and suppress tumor growth [189, 190, 191, 192, 193]. This approach exemplifies a “knifeless resection” strategy for these painful peripheral nerve sheath tumors.

In the realm of nerve repair and regeneration, biomaterial and cell‐based strategies aim to modulate the pyroptotic microenvironment. A nerve guide conduit (NGC) composed of regenerated silk fibroin loaded with conductive polymers and dimethyl fumarate (DMF) was shown to improve peripheral nerve regeneration by remodeling the intra‐conduit inflammatory milieu and suppressing Schwann cell pyroptosis [194]. Natural compounds also show promise; for instance, crocin facilitates sciatic nerve regeneration by enhancing autophagy and concurrently suppressing NLRP3/GSDMD‐dependent pyroptosis [195]. Similarly, Dental Pulp Stem Cells (DPSCs) have been reported to promote facial nerve regeneration by transferring mitochondria to Schwann cells, thereby alleviating their pyroptosis [196]. Strategies enhancing axonal repair, such as acetylglutamine (*N*‐acetyl‐l‐glutamine, NAG) [197] and epigallocatechin‐3‐gallate (EGCG) [198], also show promise. Electroacupuncture (EA), a non‐pharmacological intervention, shows clinical promise in managing diabetic neuropathy, potentially through modulating pyroptosis [199]. Nanotherapeutic approaches like nanoparticle fullerene C60 can reduce mechanical hyperalgesia and prevent disc herniation‐induced neuroinflammation [200].

Furthermore, research has expanded to other specialized tissues; in dry eye disease (DED), melatonin‐loaded liposomes were used to suppress the NLRP3/Caspase‐1/GSDMD pathway [201] or the NLRP12/NLRC4/Caspase‐4/GSDMD pathway [202], and calcitriol was applied to inhibit the NLRP3/ASC/Caspase‐1/GSDMD pathway [203]. Furthermore, targeted delivery strategies such as a prestin‐targeting peptide conjugated to Pigment epithelium‐derived factor (PrTP2‐SMP/PEDF) can inhibit NLRP3/ASC/caspase‐1‐mediated pyroptosis in spiral ganglion neurons, offering a novel approach for treating noise‐induced hearing loss [204]. These advanced modalities highlight the move toward spatially controlled, cell‐selective, and combinatorial interventions that go beyond systemic pharmacological inhibition.

### 4.6 Summary and Translational Perspective

The systematic deconstruction of pyroptosis signaling has revealed a coherent map of therapeutic targets for PN. From upstream regulators (P2X7R, CXCR4, TBK1) and core inflammasome components (NLRP3) to effector caspases (caspase‐1) and the final executors (GSDMD), each node offers a leverage point for intervention, with evidence spanning multiple etiologies of neuropathy. This review consolidates these findings into a mechanism‐centric framework, demonstrating that a single targeted agent (e.g., an NLRP3 inhibitor) holds promise across a spectrum of conditions (DPN, TN, PHN, CCI), thereby overcoming the redundancy of disease‐siloed descriptions [116, 156, 205, 206, 207, 208, 209, 210, 211, 212, 213, 214, 215, 216, 217].

This evolving paradigm positions pyroptosis as a dynamic, stage‐specific therapeutic target enabling precision modulation of neuroimmune interactions. The therapeutic strategy is two‐pronged: alleviating neuropathic pain and enhancing neural repair and regeneration. Targeting pyroptotic signaling, particularly NLRP3 inflammasome inhibition, is a groundbreaking immunopharmacological strategy for neuropathic pain [205, 206, 218, 219, 220, 221, 222, 223]. Concurrently, modulating pyroptosis in the injury microenvironment (e.g., via genetic ablation of NLRP3 [53, 224] or targeting purinergic signaling [225]) can promote structural regeneration by mitigating the detrimental effects of pyroptosis on Schwann cells and other supportive cells [186]. By integrating inhibition of inflammasome components, modulation of cytokine cascades, blockade of membrane pores, and targeting of upstream regulators, this multitiered therapeutic strategy moves beyond symptomatic relief to actively remodel the injury microenvironment. Such pyroptosis‐focused interventions concurrently alleviate neuropathic pain and promote structural regeneration, thereby addressing both functional recovery and the restoration of neuroimmune homeostasis imperatives in the treatment of PNI.

The critical translational imperative lies in navigating the context‐dependent duality of pyroptosis. The same molecular pathway that must be inhibited to treat neuropathic pain (e.g., NLRP3/caspase‐1/GSDMD) is the one that should be selectively induced to treat neuroblastoma. This necessitates a precision medicine approach. However, translating these compelling preclinical mechanisms into effective clinical therapies faces substantial challenges, which are discussed in the following section dedicated to future directions.

## 5 Challenges and Future Directions

**FIGURE 5:**
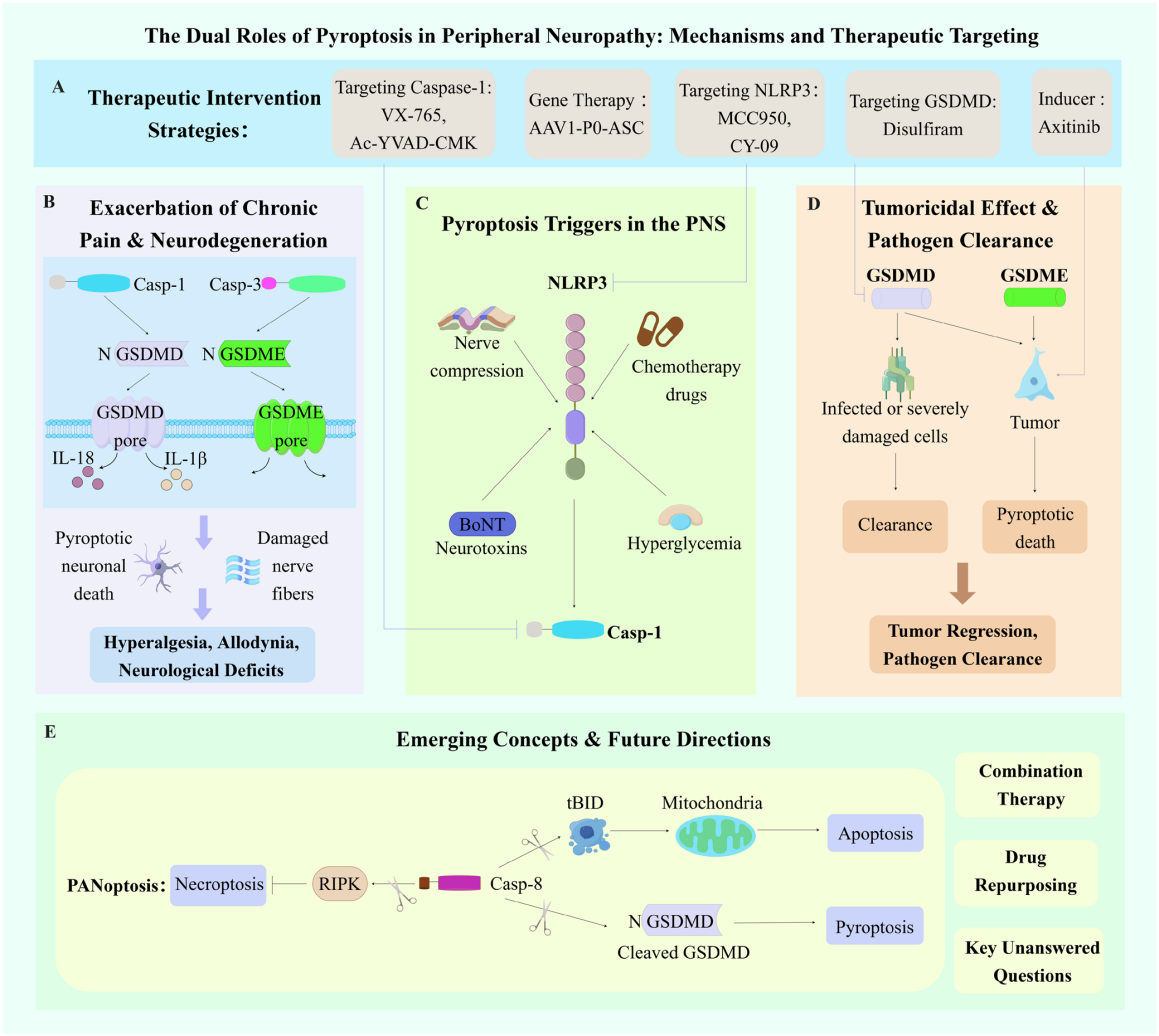
The dual roles of pyroptosis in peripheral neuropathy: mechanisms and therapeutic targeting. This schematic summarizes the mechanisms and consequences of pyroptosis in the peripheral nervous system (PNS) and potential therapeutic strategies. (A) Key therapeutic targets and agents for modulating pyroptosis, including inhibitors of Caspase‐1 (VX‐765), NLRP3 (MCC950), GSDMD (Disulfiram), and gene therapy vectors (AAV1‐P0‐ASC). (B) Pathological outcomes. Activation of the pyroptosis pathway (Caspase‐1‐GSDMD/Caspase‐3‐GSDME) in neurons promotes neuronal death, neuroinflammation, and nerve damage, leading to neuropathic pain (hyperalgesia, allodynia) and neurological deficits. (C) Triggers in the PNS, such as nerve compression, chemotherapy, neurotoxins, and hyperglycemia, which activate the NLRP3 inflammasome and caspase‐1. (D) Beneficial role in tumor clearance and host defense, where induced pyroptosis eliminates infected or malignant cells. (E) Emerging concepts, including crosstalk among PANoptosis, future therapeutic approaches like combination therapy and drug repurposing, and key unanswered questions (e.g., roles of GSDMA, GSDMB, and GSDMC).

The dual‐edged role of pyroptosis in peripheral neuropathy presents both therapeutic promise and unresolved mechanistic complexities, demanding rigorous immunopharmacological interrogation (Figure 5). While pyroptosis modulation demonstrates the potential to mitigate neuroinflammation and nerve regeneration via suppressing membrane pore formation (GSDMD/E) and IL‐1β/IL‐18 releases, its intricate crosstalk with parallel gasdermin pathways and compensatory cell death networks remains a critical knowledge gap (Figures 2, 3, 4). Strikingly, despite pyroptosis research flourishing in CNS pathologies, its regulatory significance in PN remains disproportionately underexplored, even as emerging evidence positions it as a master switch governing neuroimmune dysregulation. To bridge the gap between compelling preclinical data and clinical application, we delineate the key challenges and future directions into three interconnected themes.

### 5.1 The Dual Nature of Pyroptosis and Unmet Therapeutic Needs

Pyroptosis is known to act as a double‐edged sword in cancer, respiratory disease, inflammatory diseases, and apical periodontitis [226, 227, 228, 229, 230], conferring both protective and detrimental potential. As far as we can tell from looking through the databases, pyroptotic death is caused by the activation of the inflammasome and is generally harmful to the nervous system under most circumstances. However, in some cases, pyroptosis is beneficial to the nervous system. Axitinib‐induced Caspase‐3/GSDME pyroptosis inhibited tumor growth in the mouse neuroblastoma cell lines 9464D and 975A2 [174]. High cholesterol levels induced inflammasome activation in microglia, displayed a neuroprotective role while promoting neuronal pyroptosis [180]. This context‐dependent duality presents a fundamental therapeutic challenge: the same molecular pathway (e.g., NLRP3/caspase‐1/GSDMD) that must be inhibited to treat neuropathic pain is the one that should be strategically induced to treat neuroblastoma, necessitating a precision medicine approach. Therefore, whether pyroptosis serves as a double‐edged sword in peripheral neuropathy has not been sufficiently elucidated, and how to harness this sword to achieve better results is worth our contemplation.

Current therapeutic strategies targeting upstream inflammasomes (Caspase‐1/4/5/11, NLRP3) or downstream gasdermin effectors have shown preclinical efficacy but lack clinical validation in PNI models (Table 2). Notably, all current therapeutic strategies for PN, summarized in Table 2, focus exclusively on targeting GSDMD and GSDME pathways. To date, no known compounds specifically target GSDMA, GSDMB, or GSDMC for PN treatment, highlighting a critical unmet need in the field. Elucidating the roles of these understudied gasdermins in the PNS represents a critical future research direction. Building on our identification of the significant knowledge gaps surrounding these molecules, future studies should specifically address: Could GSDMB variants, previously associated with multiple sclerosis (MS), also be implicated in MS‐related peripheral neuropathies? Given GSDMC's emerging roles in cancer biology, might it serve as a novel therapeutic target in neuroblastoma or other PNS‐associated malignancies? What are the expression patterns and functional consequences of GSDMA in PNS homeostasis and disease? Answering these questions will be crucial for completing our understanding of the gasdermin family's role in peripheral nerve health and disease.

**TABLE 2:** Compounds/agents targeting pyroptosis‐related signaling pathways for peripheral neuropathy treatment.^a^

Compounds/Agents	Targeting the pyroptosis‐related signaling pathway	Diseases/Models	Tissues	References
A‐740003	P2X7R/NLRP3/NF‐κB/IL‐1β/and IL‐18	Myocardial infarction (MI)	Macrophage	[225]
A804598	P2X7R/P2x4R/NLRP3/and IL‐1β	Dry eye disease	Microglia	[231]
Açai berry	NLRP3/ASC/Caspase‐I/and IL‐Iβ	Diabetic peripheral neuropathy (DPN)	Spinal cord and sciatic nerve	[166]
Acetylglutamine (*N*‐acetyl‐l‐glutamine/NAG)	NLRP3/GSDMD/and caspase‐1	Brachial plexus root avulsion (BPRA)	Anterior horn of the spinal cord	[197]
ACY‐1215	HDAC6/NLRP3/NF‐κB p65/caspase‐1 p20/and IL‐18	Chronic constriction injury (CCI)	Spinal cord	[136]
Ac‐YVAD‐CMK	Caspase‐1 and GSDMD	Neuroblastoma	SH‐SY5Y	[183]
		Transected‐sciatic nerve	Schwann Cells	[171]
Alcohol	NLRP3/ASC/and caspase‐1	Experimental autoimmune prostatitis	Stromal tissues	[232]
AMD3100	CXCL12/CXCR4/NLRP3	Sciatic nerve injury	Schwann cell	[131]
AM630	CB2/NLRP3/Caspase‐1/IL‐1β/Caspase‐1 p20 and IL‐1β p17	Inflammatory pain	Skin macrophages	[206]
Amlexanox (AMX)	TBK1/NLRP3/ASC/caspase‐1/and GSDMD	Painful diabetic neuropathy	Microglia	[139]
Anthocyanin	NLRP3/Caspase‐1/IL‐1β/and TNF‐α	High salt‐induced hypertension	Peripheral sympathetic nerve activity	[154]
Aspirin‐triggered‐15‐epi‐lipoxin A4 (ATL)	NALP1/caspase‐1/and ASC	Chronic constriction injury (CCI)	Superficial laminae of the spinal dorsal horn	[158]
Axitinib	Caspase‐3/GSDME	Neuroblastoma	Cell lines 9464D and 975A2	[174]
Bay11‐7082	NLRP3/ASC/caspase‐1/IL‐1β/IL‐18/p‐IκBα/and p‐p65	Lumbar disc herniation	DRG neurons	[129]
Brilliant Blue G (BBG)	P2X7R/NLRP3/caspase‐1/and IL‐1β	Muscle pain	Muscle	[122]
		Dental inflammatory pain	Trigeminal ganglion and dorsal horn of the medulla	[31]
		Postherpetic neuralgia (PHN)	Dorsal root ganglia	[121]
		Trigeminal neuralgia (TN)	Trigeminal ganglion	[118]
BBG and MCC950	P2X7R and NLRP3	Medication overuse headache	Microglia in the trigeminal nucleus caudalis (TNC)	[120]
Botulinum toxin type A	CAMP/NLRP3/GSDMD/Caspase‐1/IL‐1/and IL‐18	Postherpetic neuralgia (PHN)	Dorsal root ganglia	[146]
Carvacrol (CRC)	Keap1/Nrf‐2/p62/NLRP3/ASC/Caspase‐1 and IL‐1β	Chronic constriction injury (CCI)	Ipsilateral sciatic nerves/DRG and spinal cord	[137]
Crocin	NLRP3/GSDMD/IL‐1β	Sciatic nerve crush injury	Sciatic nerve	[195]
Curcumin	NLRP3/IL‐Iβ	Diabetic peripheral neuropathy (DPN)	Sciatic nerve	[161]
CY‐09	NLRP3	Diabetic peripheral neuropathy (DPN)	DRG neurons	[126]
Dental pulp stem cells (DPSCs)	GSDMD/cleaved‐Caspase1/IL1β	Facial nerve injury (FNI)	Schwann cells	[196]
Dexmedetomidine	P2X4/NLRP3/and IL‐Iβ	Diabetic peripheral neuropathy (DPN)	Spines	[123]
Disulfiram or dimethyl fumarate	GSDME/caspase‐3/caspase‐9/and GSDMD	Postlingual hearing loss	Spiral ganglion neurons	[44]
Echinacoside	NLRP3/NF‐κB/IL‐1β/and IL‐18	Uremia‐induced sciatic nerve injury	Sciatic nerve	[233]
Edaravone (EDA)	NLRP3/GSDMD/Caspase‐1	Brachial plexus root avulsion (BPRA)	Motoneurons, myocutaneous nerve	[172]
Epigallocatechin gallate (EGCG)	NLRP3/Caspase‐1/GSDMD	Neuroblastoma	IMR‐32 and SK‐N‐SH cells	[73]
		Dorsal root crush injury	Spinal cord	[198]
Fullerene	NLRP3/Caspase 1/IL‐1β/substance P and CGRP	Disc herniation	DRG	[200]
Glucagon‐like peptide‐1 receptor agonist (GLP‐RA)	NLRP3/Cleaved Caspase‐1/and IL‐Iβ	Diabetic peripheral neuropathy (DPN)	Brain microglia	[162]
Glycyrrhizin (GLC)	NLRP3/TLR4/and HMGB1	Diabetic peripheral neuropathy (DPN)	Spinal cord and DRG neurons	[133]
Hydrogen‐producing silicon‐based agent	NLRP3/Caspase‐1/and GSDMD	Trigeminal neuralgia (TN)	Microglia in the trigeminal ganglion	[144]
ICI182.780	NLRP3/Caspase‐1/and GSDMD	Neuroblastoma	IMR‐32 and SK‐N‐SH cells	[73]
Imperatorin	NLRP‐3/GSDMD/caspase‐1/and IL‐18	Obesity‐induced cardiac sympathetic nerve injury	Stellate sympathetic ganglion	[124]
Jinmaitong (JMT)	TXNIP/NLRP3/Caspase‐1/GSDMC1/IL‐1β/and IL‐18	Diabetic peripheral neuropathy (DPN)	Sciatic nerves	[116, 211]
*Levo*‐tetrahydropalmatine (*l‐*THP)	Clec7a/MAPK/NF‐κB/NLRP3	Chronic constriction injury (CCI)	Spinal cord	[135]
Loganin	CXCL12/CXCR4/NLRP3/ASC/Caspase‐1	Chronic constriction injury (CCI)	Ipsilateral spinal dorsal horn	[27]
	ROS/NF‐kB/P2RX7/TNXIP/NLRP3/ASC/Caspase‐1/IL‐1β/and IL‐18	Diabetic peripheral neuropathy (DPN)	Schwann Cells	[52]
Maresin 1	NLRP3/cleaved Caspase‐1/ASC/GSDMD/IL‐1β/and NF‐κB/p65	Noncompressible lumbar disc herniation (NCLDH)	Spinal dorsal horn	[156, 157]
MCC950	NLRP3/Caspase‐1/IL‐1β and GSDMD	Activity‐induced muscle pain	Muscle hyperalgesia	[29]
		Diabetic keratopathy	Corneal	[224]
		Diabetic peripheral neuropathy (DPN)	DRG neurons	[126]
		Spared (sciatic) nerve injury (SNI)	Microglia	[221]
	NLRP3/Aβ1–42/tau protein	Trigeminal neuralgia (TN)	Cerebral cortex and hippocampus	[127]
Melatonin	NLRP3/ASC/active Caspase‐1/IL‐1β/and IL‐18	Chronic constriction injury (CCI)	Prefrontal cortex (PFC) and hippocampus (HC)	[30]
	NF‐κB/NLRP3	Neuropathic pain (NP)	Spinal nerve ligation (SNL)	[217]
Morphine	P2X7R/TLR4/HMGB1/NLRP3	Chronic constriction injury (CCI)	Ipsilateral dorsal quadrants	[207]
Nerve guide conduit (NGC) composed of regenerated silk fibroin (RSF) loaded with poly(3/4‐ethylenedioxythiophene): poly(styrene sulfonate) (P:P) and dimethyl fumarate (DMF)	NLRP3/N‐GSDMD/and cleaved‐Casp1	Sciatic nerve crush model	Sciatic nerve	[194]
*N*‐tert‐Butyl‐α‐phenylnitrone (PBN)	NLRP3/TXNIP/caspase‐1/IL‐1β/and p‐NR2B	Diabetic peripheral neuropathy (DPN)	Spinal cord	[119]
Paeoniflorin	NLRP3/ASC/caspase‐1/IL‐1β/Nrf2/and NF‐kB	Chronic constriction injury (CCI)	Spinal cord	[138]
Peptide5	NLRP3/ASC/and Caspase‐1	Chronic constriction injury (CCI)	Ipsilateral spinal cord	[61]
Pinocembrin	P2X4/Cx43/NLRP3/ASC/caspase‐1/IL‐1β/and IL‐18		Hippocampus	[141]
Pocahemiketone A	NLRP3/ASC/active Caspase‐1/GSDMD/and IL‐1β	Neuroblastoma	SH‐SY5Y	[181]
Propofol	NLRP3/ASC/Caspase‐1/p38MAPK/and NF‐kappaB	Freund's adjuvant (CFA)	Spinal cord tissues	[205]
Prestin‐targeting peptide 2 (PrTP2)‐*N*‐succinimidyl‐3‐maleimidopropionate (SMP)/pigment epithelium‐derived factor (PEDF)	NLRP3/ASC/Caspase‐1	Noise‐induced hearing loss (NIHL)	Spiral ganglion neurons (SGNs)	[204]
Salidroside	P2X7/TXNIP/NLRP3/IL‐Iβ/and IL‐18	Chronic constriction injury (CCI)	Spinal dorsal horn	[209]
Swertiamarin	NOXS/ROS/NLRP3/IL‐Iβ/and IL‐18	Diabetic peripheral neuropathy (DPN)	Spinal cord	[140]
Tangzu granule	P2X7R/NLRP3/Caspase‐1/cleaved caspase‐1/and gasdermin D (GSDMD)/and GSDMD‐N	Diabetic peripheral neuropathy (DPN)	Sciatic nerves	[115]
Tauroursodeoxycholic acid	NLRP3/Caspase‐1 p20/and GSDMD‐N	Diabetic peripheral neuropathy (DPN)	Human Schwann cells	[163]
Verapamil	TXNIP/NLRP3 and caspase‐1	Diabetic peripheral neuropathy (DPN)	DRG neurons and sciatic nerve	[130]
Vincamine	ROS/NF‐kB/and NLRP3	Diabetic peripheral neuropathy (DPN)	Dorsal root ganglia and sciatic nerve tissues	[165]
VX‐765	Caspase‐1/GSDMD/IL‐1/and IL‐18	Trigeminal neuralgia (TN)	Trigeminal nerve	[167]
Z‐DEVD‐FMK	Caspase‐3/GSDME	Neuroblastoma	SH‐SY5Y	[183]
Z‐WEHD‐FMK	Caspase‐1	Muscle pain	Muscle	[29, 122]
Z‐YVAD‐FMK	Cleaved IL‐1β and NLRP2	Freund's adjuvant (CFA) or ceramide‐induced pain	DRG neurons	[54]
	NLRP3/Caspase‐1/and GSDMD	Neuroblastoma	IMR‐32 and SK‐N‐SH cells	[73]

^a^ This table consolidates entries for each compound based on its mechanism of action. Compounds acting through an identical core signaling pathway are presented in a single row. Compounds with demonstrated, distinct mechanisms of action in different models are presented in separate rows to reflect their multi‐targeting properties.

### 5.2 PANoptosis: A Novel Conceptual Framework and Its Validation

Different forms of cell death do not function independently, even though most studies focus on a single type. Rather, there is considerable overlap and crosstalk among pyroptosis, apoptosis, necroptosis, ferroptosis, autophagy/mitophagy, and cuprotosis, allowing them to function as a network of interconnected modules [234, 235, 236, 237, 238, 239, 240, 241]. Throughout this review, we have proposed applying the PANoptosis framework to PN. This concept is presented not as an established mechanism, but as a forward‐looking hypothesis emerging from compelling yet fragmented evidence of cell death pathway crosstalk. Increasing evidence demonstrates the therapeutic potential of PANoptosis (pyroptosis, apoptosis, and necroptosis) in various diseases [5, 242, 243, 244, 245, 246, 247]. Switching among PANoptosis by Caspase‐8, caspase‐3/GSDME, caspase‐8/GSDMC, or GSDMD has been widely reported. Caspase‐8 acts as a bidirectional rheostat, modulating PANoptotic outcomes [248, 249, 250], while Caspase‐3/GSDME and Caspase‐8/GSDMC signal pathways drive apoptotic‐to‐pyroptotic switching [10, 81, 82, 251], and GSDMD facilitates pyroptosis‐necroptosis transitions under specific stimuli [74, 252, 253, 254]. It might be due to caspases that could link to PANoptosis. The recent identification of PANoptosis‐related genes in peripheral nerve injury provides an initial foundation [255], while studies in central nervous system disorders suggest the potential relevance of this pathway in neural tissues [256].

However, unresolved questions persist: what is the ratio of each kind of cell death? Whether there is a switch between or among additional cell death pathways other than PANoptosis? Can single‐cell multi‐omics decode the spatiotemporal hierarchy of PANoptotic modules in peripheral nerve injury microenvironments? Future investigations employing advanced genetic models (e.g., conditional and cell‐type‐specific multiplex knockouts), spatial transcriptomics, and in vivo real‐time cell death biosensors will be essential to validate this hypothesis. The ultimate goal is to determine whether targeting master regulators of the integrated PANoptosis machinery, rather than individual cell death pathways, could yield more effective and durable therapeutic strategies for peripheral neuropathy by preventing compensatory death pathway activation.

### 5.3 Overcoming Translational Barriers and Forging a Path Forward

Translational progress is hindered by systemic heterogeneity across neuropathy subtypes (metabolic, traumatic, vascular), overlapping death pathway activation, and a stark clinical translation gap—exemplified by the absence of trials targeting pyroptosis biomarkers (ASC specks, gasdermin cleavage) or NLRP3 antagonists like dapansutrile [257]. Reviewing the research progress on pyroptosis in peripheral neuropathy in this review will enhance our understanding of targeting pyroptosis for the diagnosis, prognosis, prevention, and treatment of peripheral neuropathy. The limitations inherent in current research models further exacerbate these barriers. First, peripheral nerve injury involves multiple diseases and various research directions. These diseases often involve all kinds of organ systems, such as diabetes and other metabolic diseases, external pressure injury, nervous system diseases, vascular diseases, etc. It is difficult to study pyroptosis in peripheral nerve injury systematically. Second, although pyroptosis pathways and related factors were identified decades ago, as a hot spot that injury is the tip of an iceberg. Third, PNS injury usually induces multiple cell death pathways simultaneously; therefore, pyroptosis alone does not fully underlie the pathological processes and the detailed regulatory mechanism of PNS injuries. Fourth, there are conflicting results on the roles of pyroptosis in peripheral neuropathy due to the complexity of the peripheral nervous system and the microenvironment during nerve injury. Furthermore, almost all cited references performed animal or cell experiments; therefore, any clinical research on the development of pyroptosis agonists or inhibitors will take a long time to fully assess the specific clinical outcome.

To break these limitations, a coordinated and phased research agenda is proposed. Mechanistic deconvolution must be prioritized by employing single‐cell and spatial transcriptomics in human nerve biopsies and advanced animal models to resolve cell‐type‐specific pyroptosis signatures. Concurrently, biomarker translation efforts should focus on validating a panel of pyroptosis indicators (e.g., plasma GSDMD‐N terminal, ASC oligomers) to enable patient stratification. Computational systems biology should be leveraged to construct predictive models of neuroimmune crosstalk, identifying key regulatory nodes within the PANoptosis network. Innovative clinical trial frameworks, such as adaptive platform trials, are necessary to efficiently evaluate combinatorial strategies (e.g., NLRP3 inhibitors paired with neuroprotective agents) and integrate molecular endpoints with patient‐centered outcomes.

Taken together, future efforts must integrate CRISPR‐based editing, nanotherapeutic delivery, and multi‐omics to map spatiotemporal gasdermin networks. Prioritizing biomarker‐stratified clinical trials and leveraging systems biology to dissect neuronal‐glial‐immune crosstalk will be critical. Decoding pyroptosis's dual roles as a neurotoxic driver and regenerative catalyst may pioneer precision therapies to recalibrate cell death dynamics, mitigate neuropathic sequelae, and restore neural homeostasis.

## Funding

This work was supported by the National Natural Science Foundation of China (32271054) and the Nantong Science and Technology Bureau (JC2023044).

## Ethics Statement

The authors have nothing to report.

## Conflicts of Interest

The authors declare no conflicts of interest.

## Supporting information


**Figure S1:** PRISMA flow diagram of literature search and study selection process.

**Appendix S1:** Full electronic database search strategies.

## Data Availability

The data that support the findings of this study are available in NCBI at https://www.ncbi.nlm.nih.gov/. These data were derived from the following resources available in the public domain: pubmed, https://pubmed.ncbi.nlm.nih.gov/?term=Pyroptosis+peripheral+nerve&size=200.
