# Supplementary material for: Pyroptosis in Peripheral Neuropathy: From Molecular Mechanisms to Therapeutic Targeting
Source: CNS Neurosci Ther. 2026 Jan 23;32(1):e70760. doi: 10.1002/cns.70760 (PMC12828674; doi:10.1002/cns.70760)
Supplement: Supplementary file 1 — Figure S1: PRISMA flow diagram of literature search and study selection process. [file CNS-32-e70760-s001.zip › FigureS1_Legend.docx]

Supplementary Figure 1. PRISMA flow diagram of the systematic literature search and study selection process.

The diagram details the identification, screening, eligibility, and inclusion stages of studies investigating pyroptosis in peripheral neuropathy. A systematic search was conducted across PubMed, Scopus, Web of Science, and Google Scholar databases for records published between January 1, 1986, and November 30, 2025. After removing duplicates, records were screened based on title and abstract, followed by a full-text assessment against predefined eligibility criteria (e.g., original research focusing on pyroptosis mechanisms in peripheral nervous system pathologies). The final number of studies included in the qualitative synthesis (narrative review) is reported.
