# Supplementary figures and images for: Pyroptosis in Peripheral Neuropathy: From Molecular Mechanisms to Therapeutic Targeting
Source: CNS Neurosci Ther. 2026 Jan 23;32(1):e70760. doi: 10.1002/cns.70760 (PMC12828674; doi:10.1002/cns.70760)

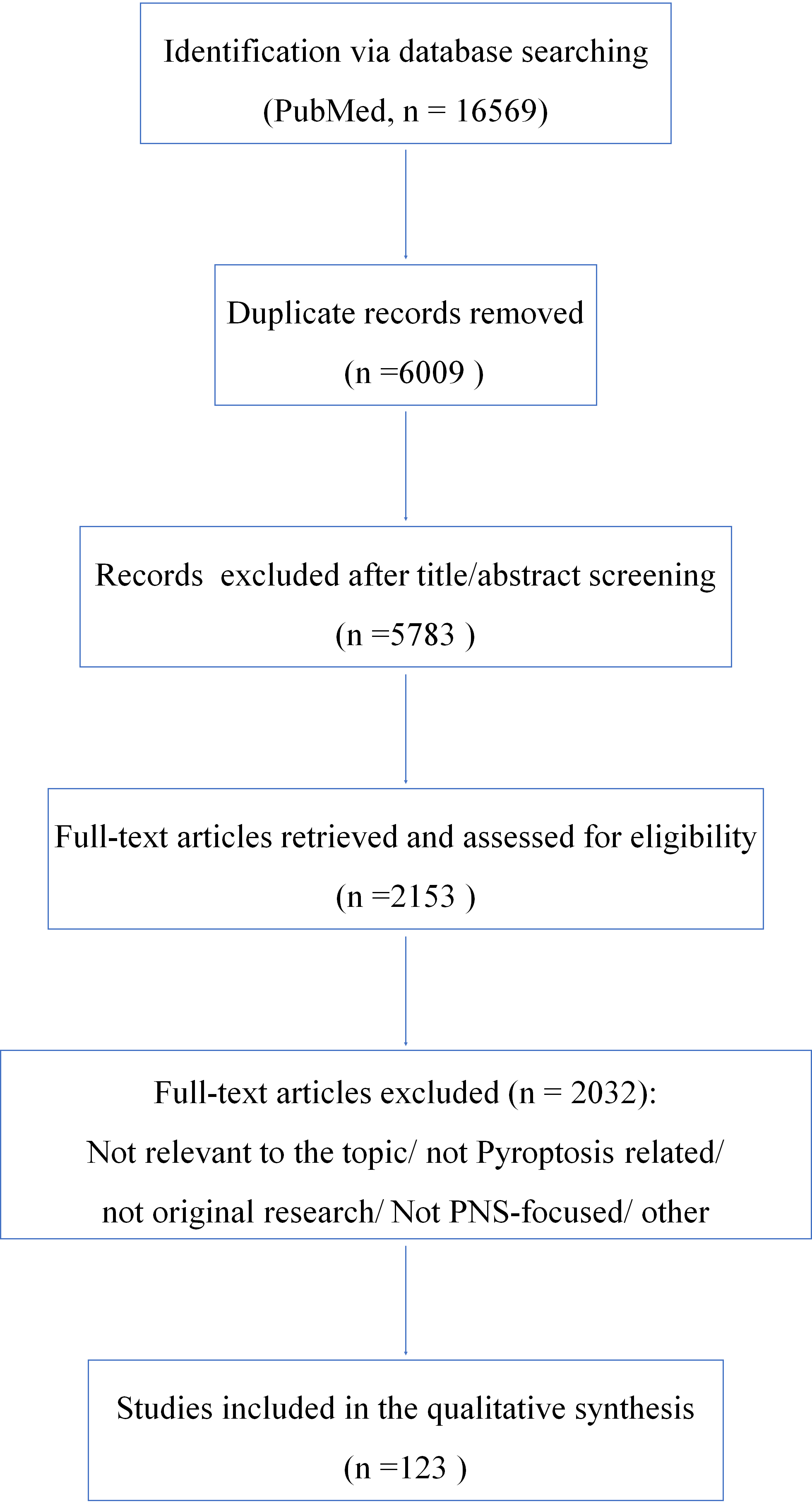

Supplement: Supplementary file 1 — Figure S1: PRISMA flow diagram of literature search and study selection process. [file CNS-32-e70760-s001.zip › cns70760-sup-0001-FigureS1.jpg]
