# Supplementary material for: Pyroptosis in Peripheral Neuropathy: From Molecular Mechanisms to Therapeutic Targeting
Source: CNS Neurosci Ther. 2026 Jan 23;32(1):e70760. doi: 10.1002/cns.70760 (PMC12828674; doi:10.1002/cns.70760)
Supplement: Supplementary file 2 — Appendix S1: Full electronic database search strategies. [file CNS-32-e70760-s002.docx]

**Literature Search Strategy**

A comprehensive and systematic literature search was performed across four major electronic databases: PubMed, Scopus, Web of Science Core Collection, and Google Scholar. The final search was conducted on Dec. 2, 2025, covering the period from January 1, 1986, to Nov. 30, 2025. To maximize both sensitivity and specificity, the search strategy employed a combination of relevant Medical Subject Headings (MeSH) terms (where applicable) and keywords/text words across the title, abstract, and keyword fields. The search was built around two core conceptual themes: “Peripheral Neuropathy” and “Pyroptosis”.

 The detailed, reproducible search syntax used for each database is provided below.

1. **PubMed Search Query:**

(

*/* Concept 1: Peripheral Neuropathy - MeSH Terms */*

(

"Peripheral Nervous System Diseases"[Mesh] OR

"Peripheral Nerve Injuries"[Mesh] OR

"Diabetic Neuropathies"[Mesh] OR

"Trigeminal Neuralgia"[Mesh] OR

"Neuralgia, Postherpetic"[Mesh] OR

"Neuroblastoma"[Mesh]

)

OR

*/* Concept 1: Peripheral Neuropathy - Keywords */*

(

(peripheral neuropath*[Title/Abstract] OR neuropath* pain[Title/Abstract]) OR

(sciatic nerve injur*[Title/Abstract] OR chronic constriction injur*[Title/Abstract] OR nerve crush[Title/Abstract]) OR

(trigeminal neuralgia[Title/Abstract] OR postherpetic neuralgia[Title/Abstract] OR neuroblastoma[Title/Abstract])

)

)

AND

(

*/* Concept 2: Pyroptosis - MeSH Terms */*

(

"Pyroptosis"[Mesh] OR

"Gasdermins"[Mesh] OR

"Inflammasomes"[Mesh] OR

"Caspase 1"[Mesh]

)

OR

*/* Concept 2: Pyroptosis - Keywords */*

(

(pyroptosis[Title/Abstract] OR pyroptotic[Title/Abstract]) OR

(gasdermin[Title/Abstract] OR GSDMA[Title/Abstract] OR GSDMB[Title/Abstract] OR GSDMC[Title/Abstract] OR GSDMD[Title/Abstract] OR GSDME[Title/Abstract]) OR

(inflammasome[Title/Abstract] OR NLRP1[Title/Abstract] OR NLRP2[Title/Abstract] OR NLRP3[Title/Abstract] OR NLRP4[Title/Abstract] OR NLRP5[Title/Abstract] OR NLRP6[Title/Abstract] OR NLRP9[Title/Abstract] OR NLRP10[Title/Abstract] OR NLRC4[Title/Abstract] OR IPAF[Title/Abstract] OR NOD1[Title/Abstract] OR NOD2[Title/Abstract] OR NAIP[Title/Abstract] OR AIM2[Title/Abstract] OR ASC[Title/Abstract] OR PYCARD[Title/Abstract]) OR

(caspase-1[Title/Abstract] OR caspase1[Title/Abstract] OR caspase-4[Title/Abstract] OR caspase4[Title/Abstract] OR caspase-5[Title/Abstract] OR caspase5[Title/Abstract] OR caspase-11[Title/Abstract] OR caspase11[Title/Abstract] OR caspase-3[Title/Abstract] OR caspase3[Title/Abstract] OR caspase-8[Title/Abstract] OR caspase8[Title/Abstract]) OR

(IL-1beta[Title/Abstract] OR IL-18[Title/Abstract])

)

)

1. **Scopus Database Search Syntax:**

TITLE-ABS-KEY(

(

"peripheral neuropath*" OR "neuropath* pain" OR

"sciatic nerve injur*" OR "chronic constriction injur*" OR "nerve crush" OR

"trigeminal neuralgia" OR "postherpetic neuralgia" OR neuroblastoma

)

AND

(

pyroptosis OR pyroptotic OR

gasdermin OR GSDMA OR GSDMB OR GSDMC OR GSDMD OR GSDME OR

inflammasome OR NLRP1 OR NLRP2 OR NLRP3 OR NLRP4 OR NLRP5 OR NLRP6 OR NLRP9 OR NLRP10 OR NLRC4 OR IPAF OR NOD1 OR NOD2 OR NAIP OR AIM2 OR ASC OR PYCARD OR

"caspase-1" OR caspase1 OR "caspase-4" OR caspase4 OR "caspase-5" OR caspase5 OR "caspase-11" OR caspase11 OR "caspase-3" OR caspase3 OR "caspase-8" OR caspase8 OR

"IL-1beta" OR "IL-18"

)

)

AND PUBYEAR > 1985 AND PUBYEAR < 2026

AND ( LIMIT-TO ( DOCTYPE , "ar" ) OR LIMIT-TO ( DOCTYPE , "re" ) ) /* Article/

1. **Web of Science Core Collection Search Syntax:**

#1: TS=("peripheral neuropath*" OR "neuropath* pain" OR "sciatic nerve injur*" OR "chronic constriction injur*" OR "nerve crush" OR "trigeminal neuralgia" OR "postherpetic neuralgia" OR neuroblastoma)

#2: TS=(pyroptosis OR pyroptotic OR gasdermin OR GSDMA OR GSDMB OR GSDMC OR GSDMD OR GSDME OR inflammasome OR NLRP1 OR NLRP2 OR NLRP3 OR NLRP4 OR NLRP5 OR NLRP6 OR NLRP9 OR NLRP10 OR NLRC4 OR IPAF OR NOD1 OR NOD2 OR NAIP OR AIM2 OR ASC OR PYCARD OR "caspase-1" OR caspase1 OR "caspase-4" OR caspase4 OR "caspase-5" OR caspase5 OR "caspase-11" OR caspase11 OR "caspase-3" OR caspase3 OR "caspase-8" OR caspase8 OR "IL-1beta" OR "IL-18")

#3: #1 AND #2

Timespan: 1986-01-01 to 2025-11-30

Document Types: Article

1. **Google Scholar Search Strategy:**

Due to the non-transparent and non-reproducible nature of Google Scholar's ranking algorithm and its lack of advanced Boolean search support, a targeted approach was employed. Searches were conducted using the following key phrase combinations to identify grey literature, preprints, and studies not indexed in the primary databases. The first 100 relevant results for each combination were reviewed.

"pyroptosis" AND "peripheral neuropathy"

"gasdermin D" AND "nerve injury"

"NLRP3 inflammasome" AND "neuropathic pain"

"pyroptosis" AND "diabetic neuropathy"

"GSDME" AND ("neuroblastoma" OR "chemotherapy")

"axon guidance conduit" AND pyroptosis

"P2X7 receptor" AND "neuropathic pain" AND inflammasome

"schwannoma" AND (pyroptosis OR inflammasome)

Additionally, to capture the latest unpublished findings, the major preprint servers **bioRxiv** and **medRxiv** were searched separately using the same core terms.

**Study Selection and Data Extraction**

Following the initial search, all retrieved records were imported into EndNote 2025 reference management software, where duplicates were removed electronically and subsequently verified manually. Records from Google Scholar and the preprint servers were screened separately and added to the pool after careful evaluation to avoid introducing duplicates of already identified records. The study selection process adhered to the Preferred Reporting Items for Systematic Reviews and Meta-Analyses (PRISMA) guidelines. The titles and abstracts of the unique records were screened for relevance, followed by a full-text assessment of the remaining articles against the inclusion criteria. Only original research articles published in English that experimentally investigated the role of pyroptosis in the context of peripheral nervous system pathologies were included for final analysis. The entire selection process, including the number of records identified, screened, assessed for eligibility, and ultimately included in the review, is summarized in the PRISMA flow diagram (Supplementary Figure 1).
